# Four-copy number alteration (CNA)-related lncRNA prognostic signature for liver cancer

**DOI:** 10.1038/s41598-022-17927-0

**Published:** 2022-08-22

**Authors:** Zhenyun Cheng, Yan Guo, Jingjing Sun, Lei Zheng

**Affiliations:** 1 Department of Clinical Laboratory, The First Affiliated Hospital of Zhengzhou University, No. 1 Jian She East Road, Zhengzhou, Henan People’s Republic of China 450052; 2 Key Clinical Laboratory of Henan Province, NO.1 Jian She East Road, Zhengzhou, Henan People’s Republic of China 450052

**Keywords:** Computational biology and bioinformatics, Biomarkers, Cancer, Tumour biomarkers, Cancer

## Abstract

The objective of this study was to identify CNA-related lncRNAs that can better evaluate the prognosis of patients with liver cancer. Prognostic molecular subtypes were identified, followed by tumor mutation and differential expression analyses. Genomic copy number anomalies and their association with lncRNAs were also evaluated. A risk model was built based on lncRNAs, as well as a nomogram, and the differences in the tumor immune microenvironment and drug sensitivity between the High_ and Low_risk groups were compared. Weighted gene co-expression network analysis was used to identify modules with significant enrichment in prognostic-related lncRNAs. In total, two subtypes were identified, *TP53* and *CTNNB1* were common high-frequency mutated genes in the two subtypes. A total of 8,372 differentially expressed (DE) mRNAs and 798 DElncRNAs were identified between cluster1 and cluster2. In addition, a four-lncRNA signature was constructed, and statistically significant differences between the Low_ and High_risk groups were found in terms of CD8 T cells, resting memory CD4 T cells, etc. Enrichment analysis showed that prognostic-related lncRNAs were involved in the cell cycle, p53 signaling pathway, non-alcoholic fatty liver disease, etc. A prognostic prediction signature, based on four-CNA-related lncRNAs, could contribute to a more accurate prognosis of patients with liver cancer.

## Introduction

Liver cancer is the most prevalent primary malignancy of the liver and the fourth leading form of life-threatening cancer worldwide^1^. Early screening and diagnosis of liver cancer, such as imaging examination and serological indicators, have been widely used and have greatly improved in recent years^2,3^. However, the early diagnosis rate of liver cancer is rather low; only 30–40% of patients are diagnosed at an early stage^4^. At present, the most effective way to treat liver cancer is radical tumor resection; nevertheless, the survival rate of patients remains poor, the 5-year survival rate being only 18%^5^. Therefore, new prognostic biomarkers are urgently needed to promote the treatment and accurate diagnosis of patients with liver cancer.

Long noncoding RNAs (lncRNAs) are RNAs that are greater than 200 nucleotides in length and lack protein-coding ability^6^. Although lncRNAs are one of the least understood classes of molecules, recent studies have shown that they are involved in a wide range of biological processes and are associated with many diseases, such as autoimmune thyroid diseases, cancer, and cardiovascular diseases^7–9^. There is evidence that abnormally expressed lncRNAs are associated with the progression of cancers^10^, including liver cancer. Wang et al. found that by activating the Wnt signaling pathway, lnctcf7 improved the self-renewal of human hepatoma stem cells^11^. Xin et al. suggested that lncRNA HULC inhibits PTEN and accelerates liver cancer through autophagy cooperation with miR15a^12^. Fu et al. illustrated that lncRNA PURPL accelerates cell proliferation in liver cancer through the regulation of p53^13^.

Copy number alteration (CNA) is a significant cause of genetic variation^14^ and defines as somatic copy number changes, which has been reported to be strongly associated with morbid consequences, such as developmental disorders and cancer^15^. There is evidence that CNA has important functions in the pathogenesis of numerous tumors^16,17^. The gain or loss of the tumor genome copy number is closely related to differential gene expression, particularly for oncogenes and tumor suppressor genes^15^. Numerous studies have reported the association between lncRNAs and CNAs in cancers. For instance, Zhong et al. have identified CNA-related lncRNAs that can better predict cervical cancer prognosis^18^, Athie et al. shown that the lncRNA ALAL-1 could be used as a regulator of lung cancer immune evasion via CNA analysis^19^, and Zhong et al. revealed the prognosis-related lncRNAs by analyzing the expression profiles of lncRNAs and CNAs in bladder cancer^20^, however, few studies have explored the regulatory relationships between lncRNAs and CNAs in liver cancer, and the CNA-related lncRNA prognostic model in liver cancer is largely unknown.

**Figure 1:**
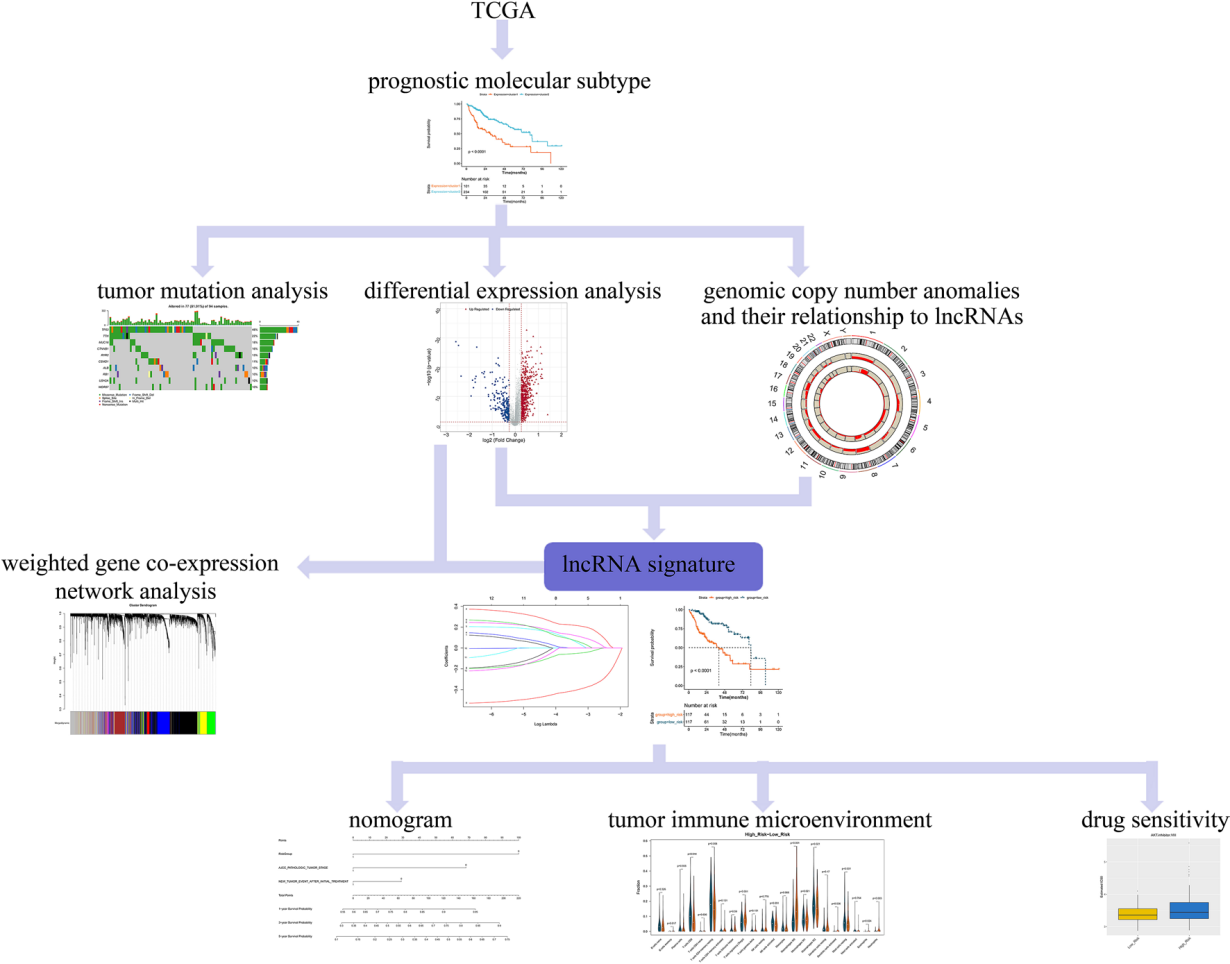
Workflow of this study.

Accordingly, the goal of the present study was to analyze the regulatory relationships between lncRNAs and CNAs in liver cancer. This was achieved by screening CNA-related lncRNAs that can evaluate liver cancer prognosis based on CNA, methylation, and gene expression data (a schematic of the study design is shown in Fig. 1). The results of this study offer predictive biomarkers for liver cancer.

## Results

### Identification of prognostic molecular subtype

**Figure 2:**
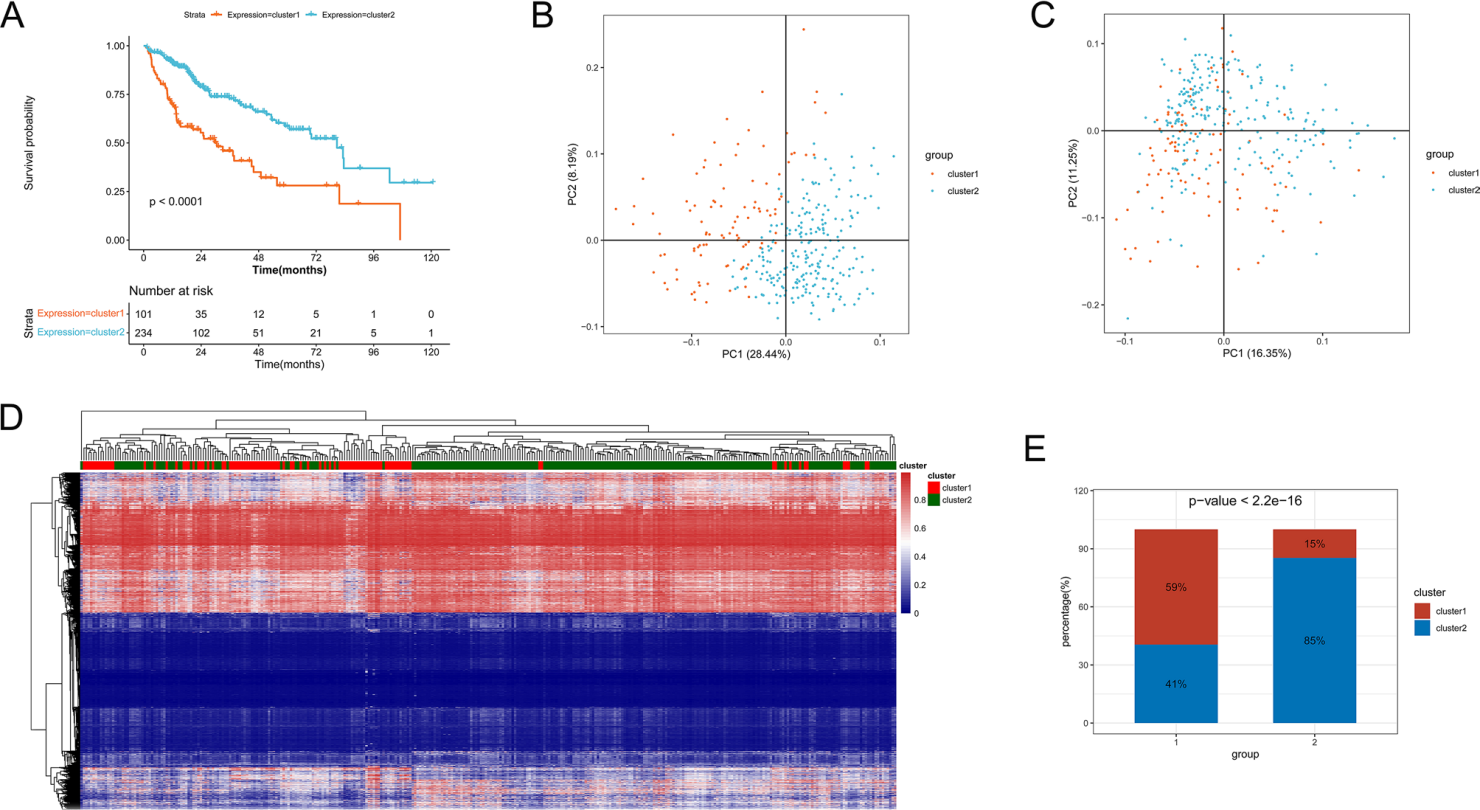
Identification of prognostic molecular subtype. (**A**): Survival and prognosis of the two subtypes. (**B**) Principal component analysis (PCA) of mRNA expression pattern, (**C**) methylation pattern. (**D**) Heatmap of the reuslts of hierarchical clustering analysis. (**E**) The differences onmethylation pattern of clusters between groups 1 and 2.

**Figure 3:**
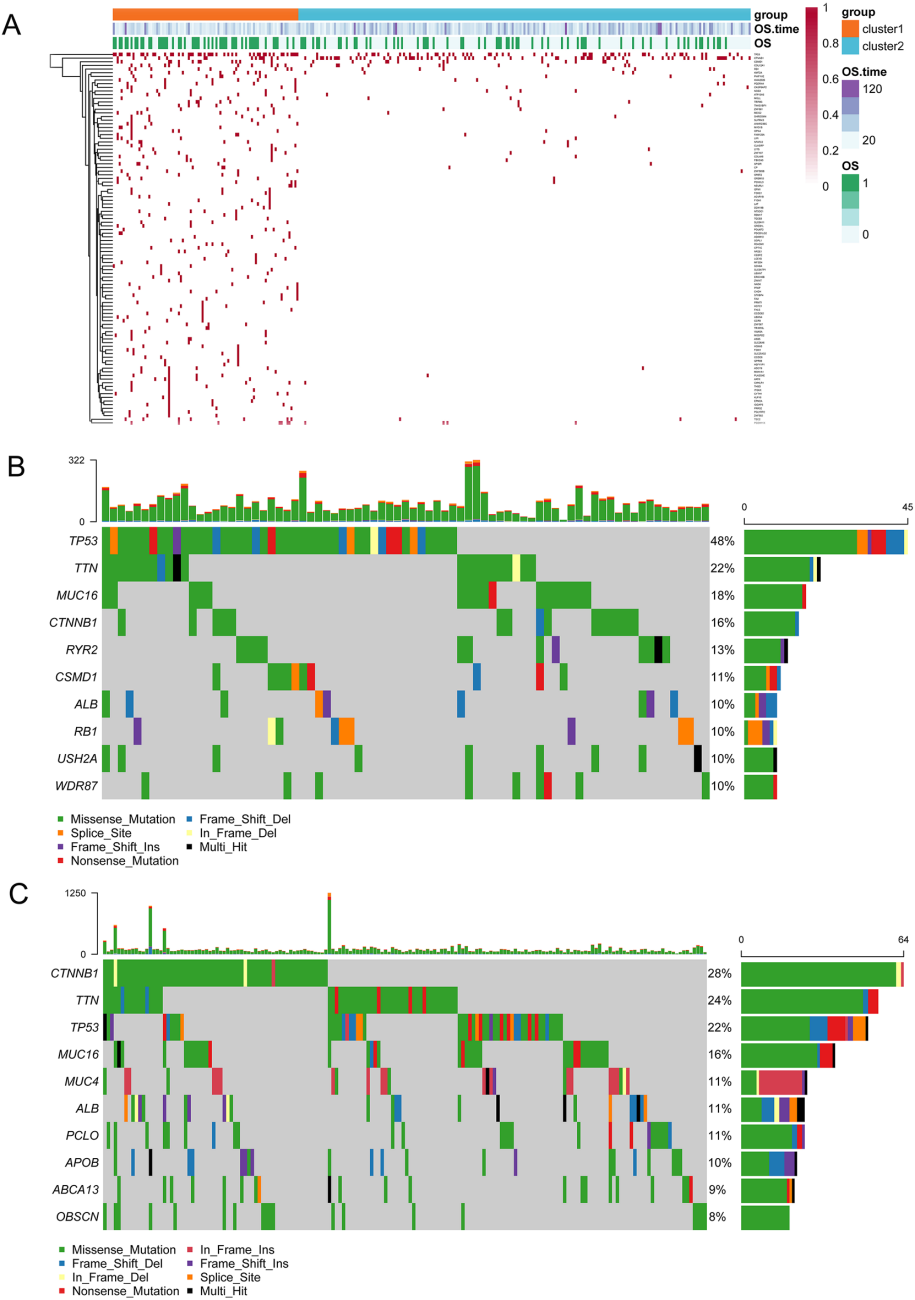
Mutation distribution of high-frequency mutation genes in the two molecular subtypes. (**A**) Heatmap of gene mutations (the gene mutations refer to SNVs only) with significant differences in mutation frequency between two subtypes. 0 indicates alive, and 1 indicates dead. Red dots indicate mutations and white dots indicate no mutations. The mutation distribution of top10 high-frequency mutation genes in (**B**) cluster1 and (**C**) cluster2. The barplot at the top indicate total number of different types of mutations in a sample.

A total of 6060 mRNAs, 3966 methylation genes, and 4961 CNA regions with significant prognostic associations were obtained. In addition, two subtypes, including cluster1 (n = 101) and cluster2 (n = 234), were identified using iClusterPlus (Table 1). Cluster2 had the most favorable prognosis (Fig. 2A). PCA results showed the mRNA expression pattern and methylation pattern in the two subtypes were different (Fig. 2B,C). Moreover, based on the methylation level values of prognostic related methylated genes in each sample, the hierarchical clustering analysis was conducted. Hierarchical clustering analysis results revealed that the samples were divided into three groups, and total 116, 218, one samples were included in 1, 2, 3 groups, respectively, and the two identified clusters were tending to cluster together (Fig. 2D). Moreover, to further observe the tendentiousness of the identified clusters contained in each group in the hierarchical clustering, the sample distribution proportion diagram of each cluster in group 1 and 2 was drawn (Fig. 2E), the results shown that group 2 was mainly included cluster2, and group 1 was mainly contained cluster1, and the methylation pattern in the two groups were found different using chi square test (P < 0.05). In addition, the frequency of 102 mutated genes showed significant differences between two subtypes (Fig. 3A; Table 2), and there were more mutated genes in cluster1. Among the 102 mutated genes, the top10 high-frequency mutated genes in subtypes are shown in Fig. 3B,C, and *TP53* and *CTNNB1* were common high-frequency mutated genes in the two subtypes.

**Table 1:** P value of the corresponding survival difference under different cluster numbers.

Cluster number	P value
2	5.05E−08
3	0.01283
4	0.349192
5	0.358615
6	0.819798
7	0.032748

**Table 2:** Mutation distribution of high-frequency mutation genes in the two molecular subtypes.

Gene	Cluster1	Cluster2	P value
TP53	0.489362	0.218341	2.51E−06
CTNNB1	0.159574	0.283843	0.027226
CSMD1	0.138298	0.061135	0.039913
COL12A1	0.117021	0.043668	0.02923
RB1	0.095745	0.030568	0.030015
KMT2A	0.085106	0.017467	0.00944
KIAA2026	0.074468	0.008734	0.003872
TSC2	0.074468	0.0131	0.011125
PIKFYVE	0.074468	0.0131	0.011125
PCDH11X	0.074468	0.017467	0.025886
MEIS2	0.06383	0.004367	0.003578
PDZRN4	0.06383	0.008734	0.012421
NOS3	0.06383	0.0131	0.032023
AATK	0.053191	0	0.002517
OR5T2	0.053191	0	0.002517
MYO1B	0.053191	0	0.002517
ANKRD36C	0.053191	0	0.002517
SHROOM4	0.053191	0.004367	0.012476
LY75	0.053191	0.004367	0.012476
NFATC2	0.053191	0.008734	0.038275
TRPM3	0.053191	0.008734	0.038275
ZNF681	0.053191	0.008734	0.038275
ATP13A5	0.053191	0.008734	0.038275
CASP8AP2	0.053191	0.008734	0.038275
MGLL	0.053191	0.008734	0.038275
TNKS1BP1	0.053191	0.008734	0.038275
SLC6A11	0.042553	0	0.009672
CYTH1	0.042553	0	0.009672
ZNF582	0.042553	0	0.009672
FOXC1	0.042553	0	0.009672
IQGAP3	0.042553	0	0.009672
NT5DC1	0.042553	0	0.009672
CMKLR1	0.042553	0.004367	0.042453
ZNF107	0.042553	0.004367	0.042453
PLA2G4E	0.042553	0.004367	0.042453
FAM129A	0.042553	0.004367	0.042453
COL4A6	0.042553	0.004367	0.042453
OPN4	0.042553	0.004367	0.042453
PCNXL3	0.042553	0.004367	0.042453
ZNF585B	0.042553	0.004367	0.042453
FBXO43	0.042553	0.004367	0.042453
LIPI	0.042553	0.004367	0.042453
ADCY9	0.042553	0.004367	0.042453
CP	0.042553	0.004367	0.042453
CLASRP	0.042553	0.004367	0.042453
SPIDR	0.042553	0.004367	0.042453
OR5M10	0.042553	0.004367	0.042453
ASB5	0.031915	0	0.037749
CCDC6	0.031915	0	0.037749
ZNF567	0.031915	0	0.037749
UBXN4	0.031915	0	0.037749
CCR9	0.031915	0	0.037749
CCDC62	0.031915	0	0.037749
EPM2A	0.031915	0	0.037749
FHL5	0.031915	0	0.037749
PGLYRP2	0.031915	0	0.037749
GPR88	0.031915	0	0.037749
SLC26A6	0.031915	0	0.037749
STXBP4	0.031915	0	0.037749
CHDH	0.031915	0	0.037749
TRIM16L	0.031915	0	0.037749
ERICH6B	0.031915	0	0.037749
RBM17	0.031915	0	0.037749
SLC9A7P1	0.031915	0	0.037749
VWA5A	0.031915	0	0.037749
PFKP	0.031915	0	0.037749
LAT	0.031915	0	0.037749
GFM1	0.031915	0	0.037749
LCE1B	0.031915	0	0.037749
FAU	0.031915	0	0.037749
HSFY1P1	0.031915	0	0.037749
DDX19B	0.031915	0	0.037749
THBD	0.031915	0	0.037749
NR2E1	0.031915	0	0.037749
TCEB3	0.031915	0	0.037749
MOSPD2	0.031915	0	0.037749
MFSD4	0.031915	0	0.037749
GREB1L	0.031915	0	0.037749
CPT1C	0.031915	0	0.037749
R3HDM1	0.031915	0	0.037749
F13A1	0.031915	0	0.037749
HCFC1	0.031915	0	0.037749
SGPL1	0.031915	0	0.037749
PRMT1	0.031915	0	0.037749
CEBPZ	0.031915	0	0.037749
PDCD1LG2	0.031915	0	0.037749
NEURL1	0.031915	0	0.037749
NADK	0.031915	0	0.037749
FOXI1	0.031915	0	0.037749
ADAM10	0.031915	0	0.037749
PRR32	0.031915	0	0.037749
ACVR1B	0.031915	0	0.037749
UBXN7	0.031915	0	0.037749
SLITRK3	0.031915	0	0.037749
MAN1A1	0.031915	0	0.037749
SCN8A	0.031915	0	0.037749
KLF16	0.031915	0	0.037749
ITGAX	0.031915	0	0.037749
HOXA3	0.031915	0	0.037749
ZWINT	0.031915	0	0.037749
SLC25A32	0.031915	0	0.037749
POU6F2	0.031915	0	0.037749

### Identification of differentially expressed (DE) mRNAs and DElncRNAs in two subtypes

Applying the screening criteria of P < 0.05, and |log_2_FC|> 0.263, a total of 8,372 DEmRNAs (7,123 up-regulated and 1249 down-regulated) and 798 DElncRNAs (577 up- and 221 down-regulated) were identified between cluster1 and cluster2 (Supplementary Fig. 1A,B).

### LncRNAs abnormal expressions related to CNAs

**Figure 4:**
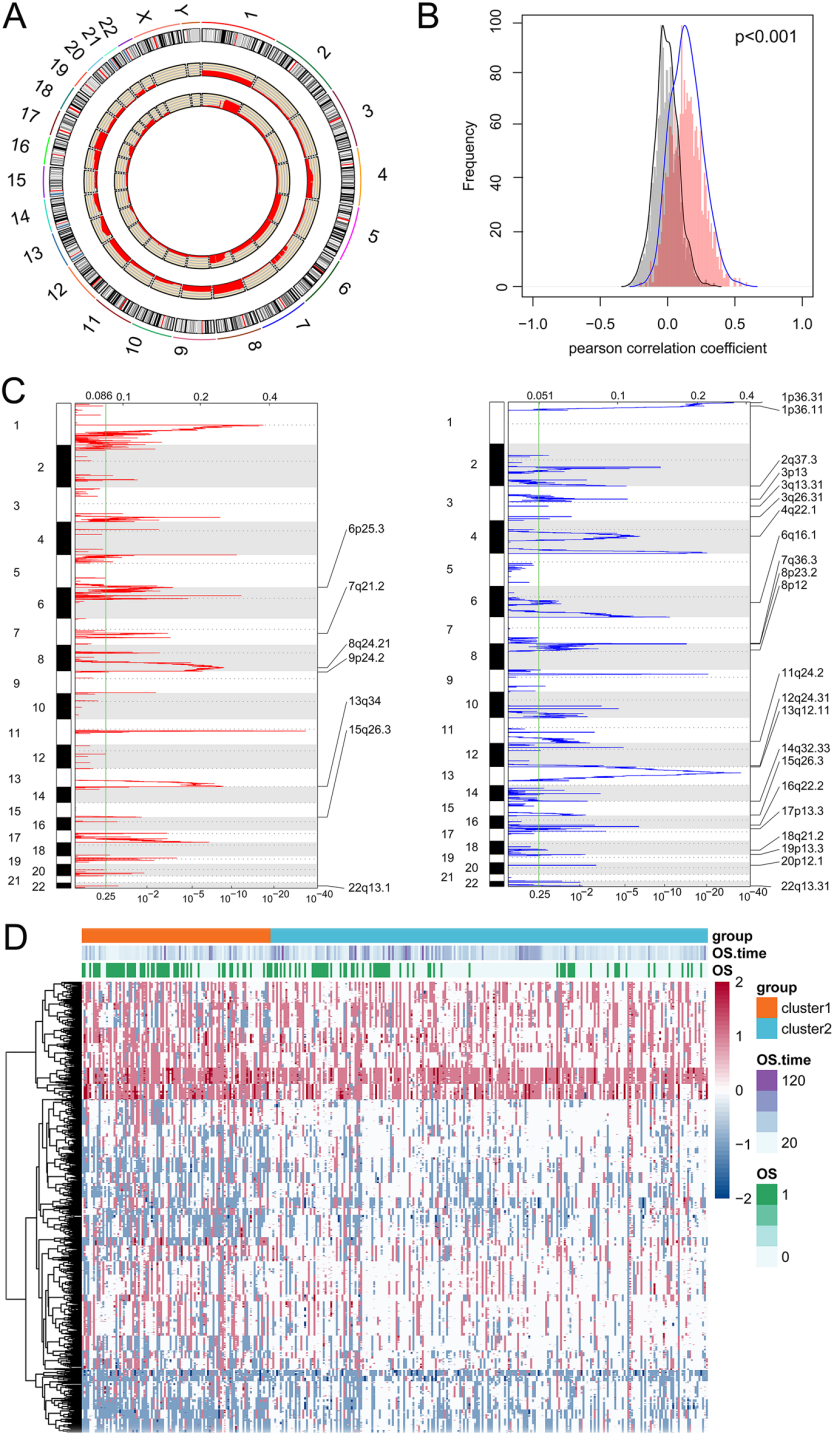
Genomic copy number anomalies and their relationship to lncRNAs. (**A**) Distribution of lncRNA copy number gain and loss in the genome. The innermost layer indicates copy number gain, the second layer indicates copy number loss, and the red height indicates variation frequency. (**B**) The correlation distribution between lncRNA expressions and copy number variation (CNAs), grey represents the distribution under random conditions, orange represents the distribution under actual conditions. (**C**) The lncRNAs located in the focal CNA peaks. False-discovery rates and scores from GISTIC 2.0 for alterations (x-axis) are plotted against genome positions (y-axis); dotted lines indicate the centromeres. The losses (right, blue) and gains (left, red) of lncRNAs genes are also shown. (**D**) Heatmap of CNA in lncRNA. Blue dot indicates loss, red dot indicates gain, and white dot indicates no variation.

The variant frequency of lncRNAs in the samples was calculated to evaluate the association between CNAs and lncRNA expression. The frequency of copy number gains and losses of lncRNAs on each chromosome varied (Fig. 4A); for example, numerous copies of chromosomes 4, 8, 9, and 17 were deficient, whereas there were a greater number of copies of chromosomes 5, 6, and 7. In addition, based on the expression profile and CNA profile of 1,358 lncRNAs, the correlation distribution between the copy number and lncRNA expression profile showed an overall trend of positive association (Fig. 4B). Numerous regions with lncRNA copy number gain and loss were revealed (Fig. 4C), indicating that the abnormal lncRNAs copy number might be associated with the progression of liver cancer. In addition, as shown in the heatmap (Fig. 4D), the variant ratio of lncRNAs in cluster1 increased compared to that in cluster2, and the Chi square test results shown that among the 1,358 lncRNAs, total 1,238 lncRNAs had significant difference on CNA between cluster1 and cluster2 (Supplementary Table 1). Next, a total of 52 lncRNAs with CNA frequency > 75% in samples were screened, and the differences in the expression of these 52 lncRNAs with copy number gain, copy number loss, and normal copy number were evaluated using Kruskal–Wallis test. The results showed that the expression of most lncRNAs were significantly difference among the three groups (P < 0.05; Supplementary Fig. 2 and Supplementary Table 2), suggesting that lncRNA abnormal expressions are associated with CNAs.

### Establishment of a lncRNA signature

**Figure 5:**
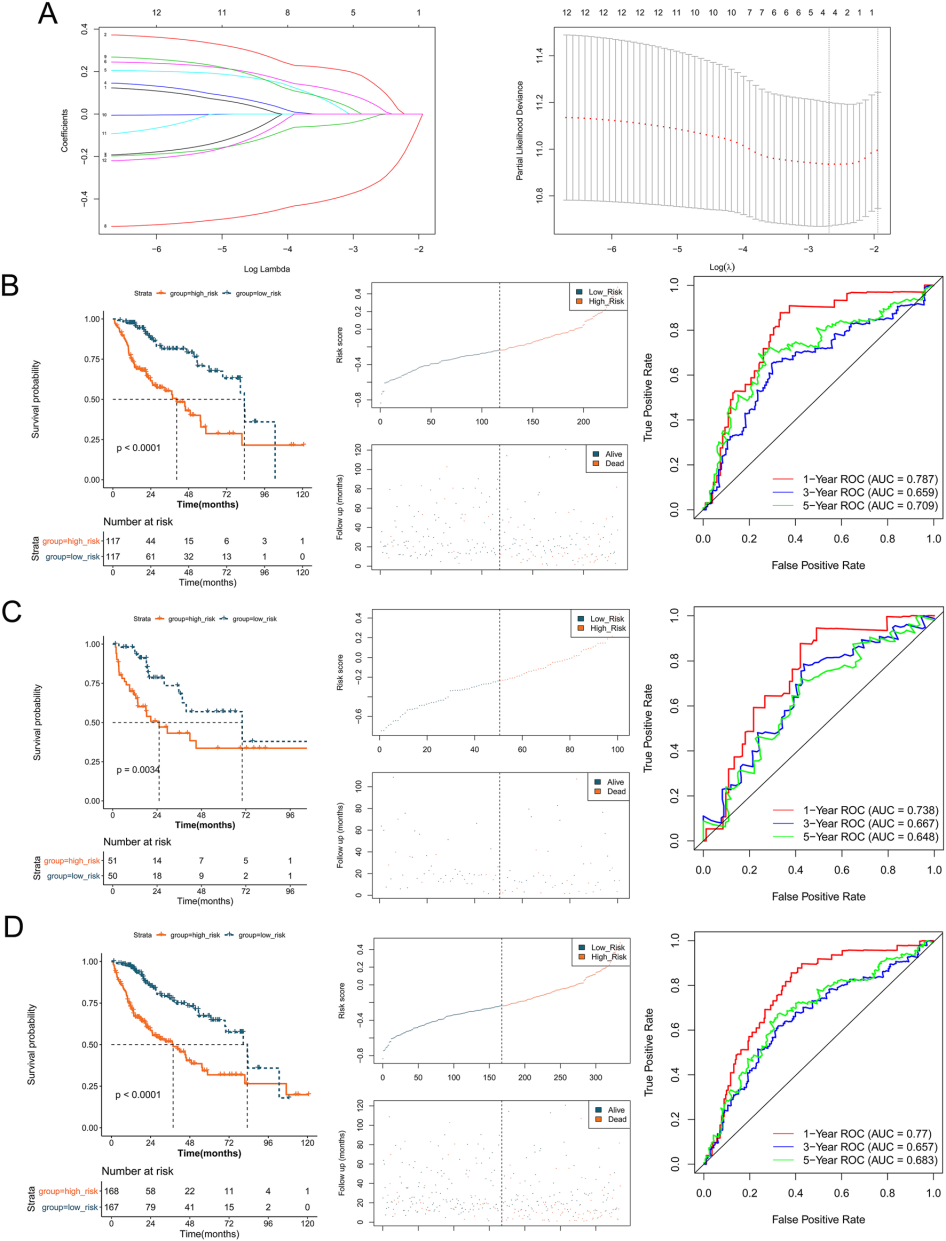
Identification of lncRNA prognostic markers with abnormal copy number and establishment of lncRNA signature. (**A**) LASSO Cox regression analysis. The left vertical line in the plot shows the CV-error curve hits its minimum. The right vertical line shows the most regularized model with CV-error within 1 standard deviation of the minimum. Kaplan–Meier survival analysis, plots of risk scores distribution, time-dependent receiver operating characteristic (ROC) analysis for the (**B**) training set, (**C**) testing set, and (**D**) validation set.

**Figure 6:**
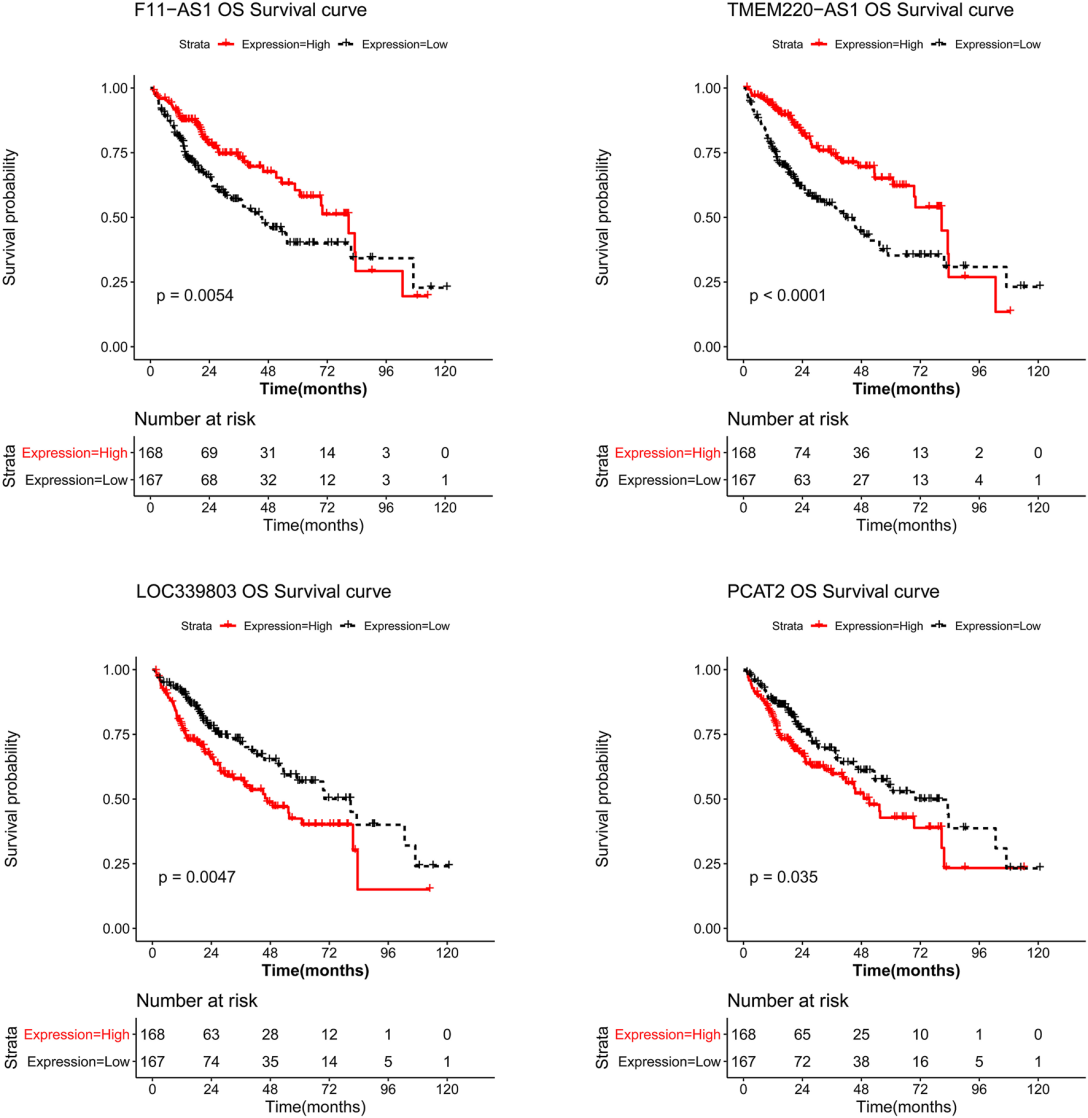
Kaplan–Meier survival analysis of four lncRNAs in the signature.

A total of 34 lncRNAs were screened as candidate lncRNAs, as described in the Methods section. Univariate Cox regression analysis was conducted, and a total of 12 prognostic-related lncRNAs were identified (Table 3). LASSO Cox regression analysis was performed, and four lncRNAs were utilized to build the signature (Fig. 5A), containing LOC339803, F11-AS1, PCAT2, and TMEM220-AS1. The prognostic capacity of the lncRNA signature was also evaluated in training, testing, validation sets, and the patients in the High_risk group had a poorer prognosis than those in the Low_risk group (Fig. 5B–D). The AUCs at 1-, 3- and 5-year survival time were all approximately 0.7 (Fig. 5B–D). In addition, patients with high expression of F11-AS1 and TMEM220-AS1 had a favorable prognosis, whereas high expression of LOC339803 and PCAT2 indicated poor prognosis (Fig. 6).

**Table 3:** Univariate Cox regression results.

LncRNA	HR	Lower.95	Upper.95	P value
TMEM220-AS1	0.475	0.326	0.691	1.039E−04
LOC101927151	1.814	1.256	2.619	1.498E−03
SNHG16	1.702	1.176	2.464	4.806E−03
LOC339803	1.685	1.169	2.430	5.206E−03
F11-AS1	0.598	0.414	0.863	5.972E−03
SVIL-AS1	1.654	1.151	2.377	6.547E−03
UBR5-AS1	1.621	1.122	2.340	1.003E−02
RAB11B-AS1	0.636	0.443	0.914	1.456E−02
TSTD3	1.568	1.091	2.254	1.509E−02
ZFAS1	1.516	1.055	2.179	2.460E−02
PCAT2	1.475	1.026	2.121	3.576E−02
LOC101929147	1.462	1.017	2.102	4.009E−02

### Construction of the nomogram

**Figure 7:**
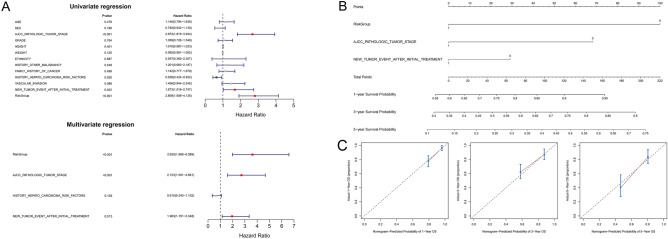
Univariate and multivariate Cox analysis of the signature combined clinical features and construction of the nomogram. (**A**) Forest characteristics of clinical features and risk score using univariate and multivariate Cox analysis. (**B**) Construction of the nomogram. For each patient, three lines are drawn upward to determine the points received from the three predictors (RiskGroup, AJCC_PATHOLOGIC_TUMOR_STAGE, and NEW_TUMOR_EVENT_AFTER_INITIAL_TREATMENT) in the nomogram. The nomogram is applied by adding up the points identified on the points scale for each variable to a total points amount. The sum of these points is located on the ‘Total Points’ axis. Finally, beneath the total points, the probability of 1-, 3-, 5-year overall survival is projected on the bottom scales. (**C**) The calibration plot for validation of the nomogram. The Y-axis represents actual survival, and the X-axis represents nomogram-predicted survival.

After the univariate Cox regression analysis was carried out, RiskGroup, AJCC_PATHOLOGIC_TUMOR_STAGE, NEW_TUMOR_EVENT_AFTER_INITIAL_TREATMENT were identified with P < 0.05 (Fig. 7A), and these characteristics were used to build a nomogram (Fig. 7B). The calibration curves were matched to actual 1-, 3-, and 5-year survival (Fig. 7C).

### Clinical characteristics

The distribution of each clinical characteristic in the High_ and Low_risk groups was statistically explored, and the results showed significant differences in Pathologic-T, Pathologic-stage, Grade, Vascular invasion between the Low_ and High_risk groups (Table 4). In addition, the High_risk group presented more cluster1 samples (Supplementary Fig. 3), which might explain the poor prognosis of patients in the High_risk group.

**Table 4:** Clinical features of the dataset.

Characteristics total cases	N of case 335	Riskgroup		P value
		Low_risk	High_risk	
**Age (years)**				
< 65	200	100	100	0.440
≥ 65	135	67	68	
**Gender**				
Male	230	118	112	0.503
Female	105	49	56	
**Pathologic M**				
M0	238	119	119	1.000
M1	3	1	2	
MX	94	47	47	
**Pathologic N**				
N0	236	114	122	0.183
N1	2	0	2	
NX/NA	97	53	44	
**Pathologic T**				
T1	166	96	70	0.015
T2	84	36	48	
T3	70	27	43	
T4	12	5	7	
TX/NA	3	3	0	
**Pathologic stage**				
Stage I	159	91	68	0.038
Stage II	77	35	42	
Stage III	75	28	47	
Stage IV	3	1	2	
NA	21	12	9	
**Grade**				
G1	50	34	16	0.004
G2	158	78	80	
G3	110	49	61	
G4	12	2	10	
NA	5	4	1	
Height	167.7 ± 9.1	168 ± 8.7	167.5 ± 9.5	0.665
Weight	73.1 ± 19.1	74.2 ± 19.1	72.1 ± 19.2	0.321
**Ethnicity**				
Hispanic or Latino	15	5	10	0.382
Not Hispanic or Latino	304	153	151	
NA	16	9	7	
**History other malignancy**				
Yes	30	19	11	0.175
No	305	148	157	
**Family history of cancer**				
Yes	102	53	49	0.705
No	190	91	99	
NA	43	23	20	
**History hepato carcinoma risk factors**				
Yes	238	120	118	0.747
No	80	38	42	
NA	17	9	8	
**Vascular invasion**				
Yes	100	48	52	0.032
No	184	102	82	
NA	51	17	34	
**New tumor event after initial treatment**				
Yes	92	47	45	0.056
No	154	85	69	
NA	89	35	54	

### Tumor immune microenvironment

**Figure 8:**
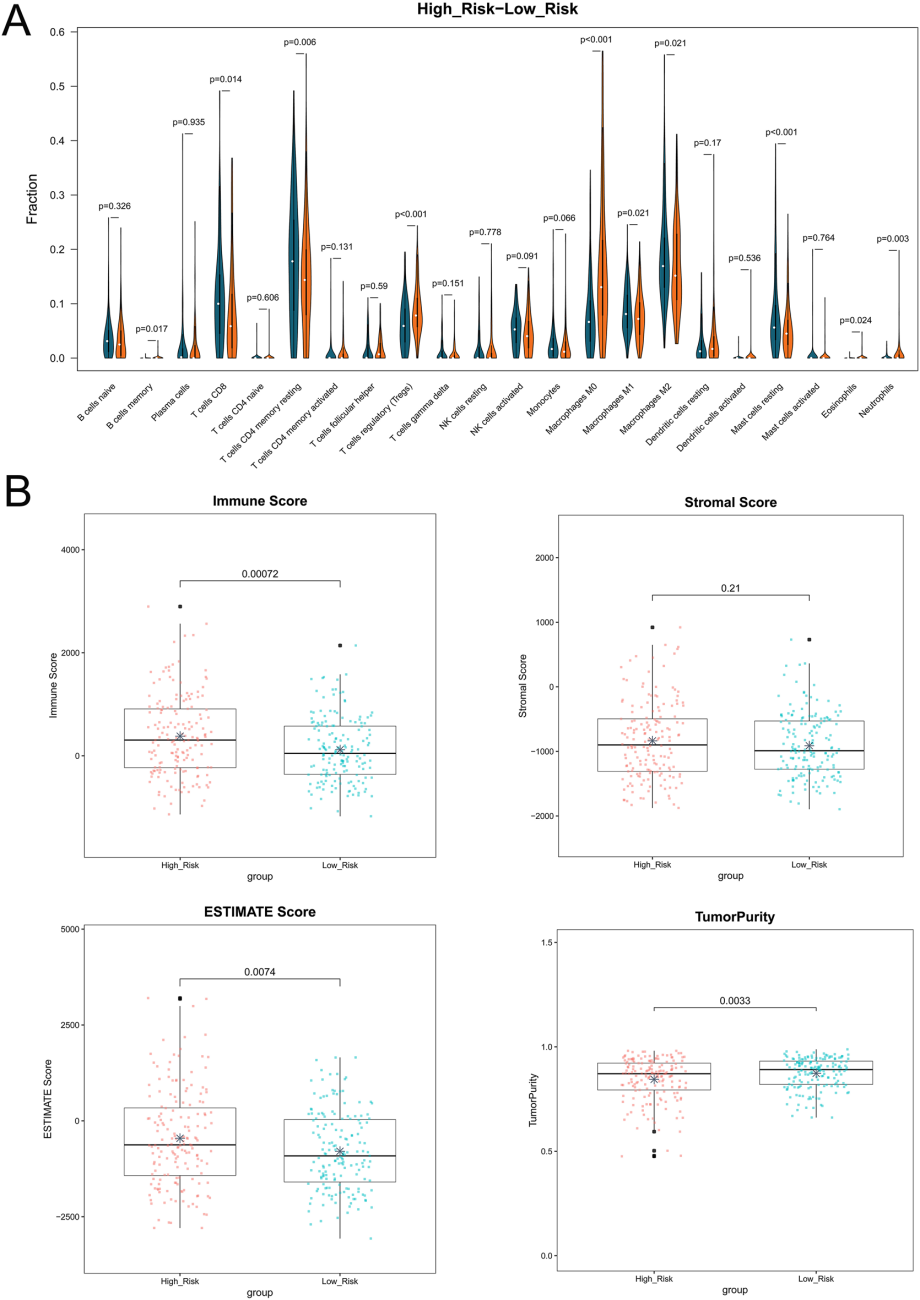
Tumor immune microenvironment. (**A**) The difference of tumor-infiltrating immune cells between risk groups. (**B**) The difference of immune score, stromal score, estimate score and tumor purity between risk groups. The stars in the boxplots represent mean value.

As shown in Fig. 8A, ten immune cells, including memory B cells, regulatory T cells (Tregs), and M0 macrophages, showed obvious differences between the Low_ and High_risk groups. There were also statistically significant differences in immune score, estimate score, and tumor purity between the Low_ and High_risk groups (Fig. 8B).

### Drug sensitivity prediction

The IC50 of 138 drugs was quantified, and the differences between High_ and Low_risk groups were compared. In the case of 30 of these drugs (including Erlotinib, Lapatinib, and Gefitinib), a significant difference in IC50 was found between the two groups (Supplementary Fig. 4; Table 5), suggesting that the High_risk group may be more resistant to these drugs.

**Table 5:** The IC50 of 30 drugs.

Drug	P value
GW.441756	2.32E−15
Erlotinib	2.57E−13
CCT007093	3.33E−13
BMS.708163	2.71E−12
Lapatinib	5.77E−11
Gefitinib	1.38E−09
AMG.706	9.53E−09
Imatinib	1.52E−07
Nutlin.3a	4.16E−07
PD.0332991	4.75E−07
Roscovitine	9.33E−07
AZD.0530	1.14E−06
KIN001.135	1.90E−06
Bicalutamide	6.27E−05
Axitinib	0.000353
AZD6244	0.000508
EHT.1864	0.000775
Metformin	0.000964
LFM.A13	0.001183
WO2009093972	0.001322
PD.0325901	0.001619
GNF.2	0.001878
AKT.inhibitor.VIII	0.003844
MG.132	0.00448
DMOG	0.004832
OSI.906	0.010286
Bryostatin.1	0.012467
CI.1040	0.016188
VX.702	0.029052
PF.02341066	0.03113

### Identification of enriched lncRNA modules

**Figure 9:**
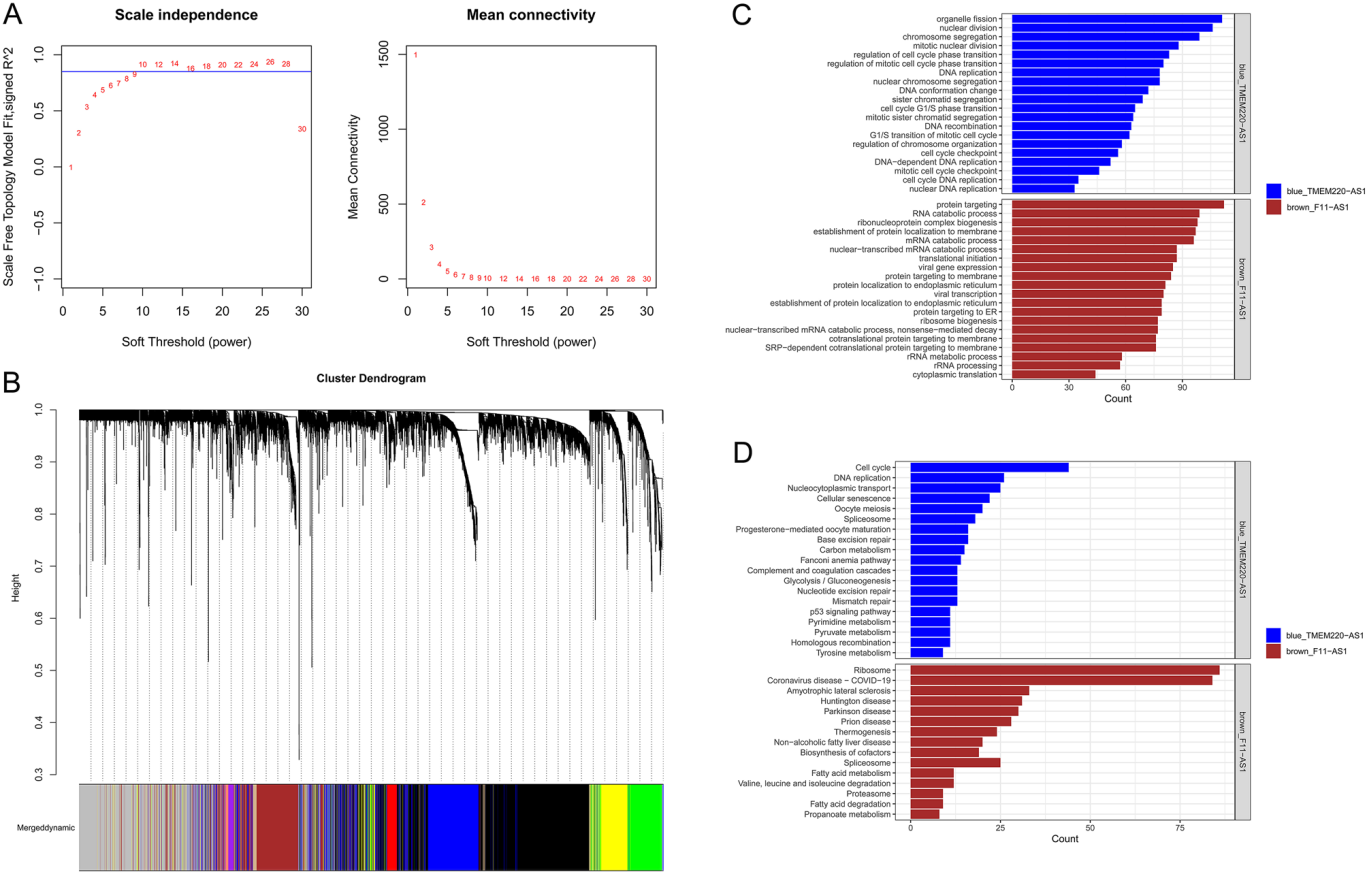
Co-expression modules of prognostic-related lncRNAs and differentially expressed (DE) mRNAs. **(A**) Determination of soft threshold for adjacency matrix. The horizontal axis represents the soft threshold power and the vertical axis represents the square of the correlation coefficient of between log_2_k and log_2_p (k). The blue line indicates where the correlation coefficient is 0.85, and the corresponding soft threshold power is 10. (**B**) Gene dendrogram derived from hierarchical clustering. Different modules are indicated by colors underneath the dendrogram. (**C**) Gene Ontology (GO)-biological process (BP) and (**D**) Kyoto Encyclopedia of Genes and Genomes (KEGG) pathways involved in the blue and brown module.

The WGCNA package was employed to build a scale-free co-expression network, and the soft threshold power for matrix transformation was analyzed with the square of the related coefficient between log2k and log2p (k) being 0.85, and the power = 10 (Fig. 9A). For each module, the minimum number of genes was set to 30, and the similarity was greater than 0.1. These modules were clustered, and the modules with correlation coefficients greater than 0.8 were merged, yielding a total of seven modules (Fig. 9B). The two lncRNAs were clustered into a gray module, and the other two lncRNAs, TMEM220-AS1 (blue module) and F11-AS1 (brown module), were further analyzed. The enrichment analysis showed that the blue module was enriched in 487 GO-BP terms and 19 KEGG pathways (including cell cycle, p53 signaling pathway, DNA replication), and the brown module was enriched in 168 GO-BP terms and 15 KEGG pathways (including non-alcoholic fatty liver disease, fatty acid degradation, etc.) (Fig. 9C,D).

## Discussion

CNAs have important functions in tumor progression^21^. In the present study, iClusterPlus was utilized for cluster analysis based on mRNA expression, methylation, CNA data, and iClusterplus, which can decrease the dimension of a dataset without altering the sample size. The results showed that two subtypes, cluster1 and cluster2, were identified. Cluster2 had the most favorable prognosis, and the CNA frequency of lncRNAs in cluster1 was higher than that in cluster2, which suggests that CNA-related lncRNAs were correlated with the prognosis of patients with liver cancer. Moreover, *TP53* and *CTNNB1* were common high-frequency mutated genes in both subtypes. In cancer, *TP53* is the most frequently mutated gene, and more than 50% of human tumors carry *TP53* gene mutations, including liver cancer^22–24^. Mutations in *CTNNB1* have been implicated in the pathogenesis of liver cancer^25^. These results indicate that subtype classification might help evaluate the prognosis of patients with liver cancer and have specific regulatory relationships at the level of transcription, genome, and epigenome.

Zheng et al. aimed to screen prognostic biomarkers of lncRNA associated with CNA in ovarian cancer^26^, however, prognostic biomarkers of four lncRNAs associated with CNA were screened after LASSO Cox regression analysis in this study, containing LOC339803, F11-AS1, PCAT2, and TMEM220-AS1, and these four lncRNAs were further used to build the CNA-related lncRNA prognostic model for liver cancer. The patients in High_risk group had a poorer prognosis than patients in Low_risk group in all sets. The AUCs at 1-, 3-, and 5-year survival times in all sets were all approximately 0.7, suggesting that the performance of the lncRNA signature was reliable. In addition, patients with high expression of F11-AS1 and TMEM220-AS1 had a favorable prognosis, whereas high expression of LOC339803 and PCAT2 was associated with poor prognosis. Du et al. found that lncRNA F11-AS1 regulates PTEN expression by competitive binding with miR-3146 and inhibits the progression of liver hepatocellular carcinoma, and F11-AS1 may be used as a therapeutic target for liver hepatocellular carcinoma^27^. Cao et al. revealed that TMEM220-AS1 inhibits hepatocellular carcinoma by regulating the miR-484/MAGI1 axis^28^. Xue et al. documented that lncRNA LOC339803 facilitates the invasion and migration of hepatocellular carcinoma cells by acting as a ceRNA of miR-30a-5p^29^. Han et al. implied that PCAT2 plays a vital role in prostate cancer^30^. Our results are consistent with those reported above. However, few studies have reported PCAT2 expression in liver cancer. In addition, enrichment analysis showed that TMEM220-AS1 is involved in the cell cycle, DNA replication pathways, p53 signaling pathway, etc., and F11-AS1 is involved in non-alcoholic fatty liver disease, fatty acid degradation pathways, etc. The cell cycle is a complex process that is regulated by a variety of proteins at multiple levels, and the cell cycle pathway plays a crucial role in tumorigenesis^31^. Studies have reported that the p53 signaling pathway plays a vital role in the regulation of tumor progression^32–34^.

DNA replication is a basic biological process, in this process, disorder can lead to genomic instability, which is a hallmark of cancer^35^. Non-alcoholic fatty liver disease can develop into cirrhosis via fibrosis and can be complicated by hepatocellular carcinoma^36,37^. As fatty acids are essential for cancer cell proliferation, fatty acid degradation could provide a therapeutic strategy^38^. Thus, LOC339803, F11-AS1, PCAT2, and TMEM220-AS1 might have vital functions in the pathogenesis of liver cancer and could be used as prognostic markers for the cancer.

Because changes in the immune microenvironment have a profound effect on the progression of liver cancer^39^, the immune microenvironment changes were analyzed using the ESTIMATE algorithm and CIBERSORT. The results revealed that ten immune cells, and the immune, estimate scores, and tumor purity had obvious differences between Low- and High_risk groups. It has been reported that in hepatocellular carcinoma, regulatory T cells (Tregs) and exhausted CD8 T cells are increased and may clonally expand^40^. In patients with liver cancer, tumor-associated macrophages (TAMs) are regularly increased through immunohistochemical staining^41^. Rohr-Udilova et al. found that resting mast cells in hepatocellular carcinoma were increased when compared to healthy livers^42^. In addition, high immune and estimate scores are correlated with clinicopathological characteristics and poor prognosis in cancer^43^. In addition, statistically significant differences in IC50 of 30 drugs were found when Low- and High_risk groups were compared, among these drugs were Erlotinib, Lapatinib, Gefitinib, etc. These results revealed that these CNA-related lncRNA signatures might better predict the survival of patients with liver cancer, and these ten immune cells are related to the progression of liver cancer.

However, this study had some limitations. First, the data analyzed were downloaded from public databases, and external validation was required to show the utility of lncRNAs related signatures. Second, the fold-change was not calculated in the drug sensitivity prediction anlaysis dut to no quantized value that can represent resistant or sensitive, and further research should be conducted. Besides, the lncRNAs from which short peptide are transcribed should be considered in this study. In addition, the immune cell proportion was estimated using only the CIBERSORT algorithm, further relevant experiments should be carried out to verify this. Moreover, that’s would be better if there were more relevant experiments to validate the biomarkers and pathways identified in this study.

## Conclusion

In summary, a CNA-related lncRNA prognostic signature, which is closely correlated with the immune microenvironment, was constructed in this study. This signature is likely to improve the accuracy of liver cancer prognosis and provide insights into predictive biomarkers or potential targets for patients with liver cancer.

## Materials and methods

### Data collection and processing

The gene expression RNA-seq (log2(fpkm + 1)) data of GDC TCGA LIHC were downloaded from the UCSC Xene platform (https://xenabrowser.net/)^44^, and the genes with expression less than 1 in more than half of the samples were filtered out. Those genes with “protein_coding” annotation (based on the downloaded gene annotation file in GENCODE V22 version) were reserved as mRNA, and the genes with “antisense,” “sense_intronic,” and “lincRNA”, etc., annotation information were reserved as lncRNA. In addition, the CNA (cna_hg19.seg; https://cbioportal-datahub.s3.amazonaws.com/lihc_tcga.tar.gz; Affymetrix SNP 6.0 array), 450k methylation (gene level methylation values, and the probe with the most obvious negative correlation with the gene was selected as the methylation value of the gene, so that each gene has a methylation level value), and clinical and survival information in the TCGA database were obtained from the cBioportal website (http://cbioportal.org)^45^. The samples corresponding to RNA-seq, CNA, 450K methylation, and clinical survival information (OS and OS.time) were matched one by one, and the samples with these data were retained. As a result of these screenings, a total of 335 samples meeting the requirements were obtained; the clinical features of these samples are shown in Table 4.

### Screening of prognostic molecular subtype

Based on the mRNA expression, 450k methylation, CNA, and survival information of the 335 samples, the FsbyCox function in the CancerSubtypes package^46^ was employed to perform the univariate Cox regression analysis, and the prognostic-related characteristics of mRNA, methylation gene, and CNA region were acquired with the cutoff value of P < 0.05. Cluster analysis was then carried out using iClusterPlus^47^ in R software package, and the parameter was set as K = 1:6 in order to select the best number of clusters. Combined with the sample survival information, the log-rank test was conducted, and the classification results with the lowest P value were selected to determine the molecular subtypes. To verify the classification results, principal component analysis (PCA) and hierarchical clustering analysis were performed.

### Tumor mutation analysis

The somatic mutation file processed using Mutect software was obtained from the TCGA database^48^. The oncoplot function in maftools^49^ R package was employed to draw the waterfall of the top10 mutated genes with a high mutation frequency. The mutation frequency of each gene in different subtypes was calculated, and the differences were compared using the Chi-square test.

### Differential expression analysis

The linear regression and empirical Bayesian methods offered in the limma package^50^ in R software were utilized to conduct differential expression analysis, and the P values were adjusted using the Benjamini & Hochberg method for multiple comparisons. The DEmRNA and DElncRNA were screened with a cutoff value of P < 0.05 and |log_2_FC|> 0.263 owing to acquiring more DElncRNA for subsequent analysis.

### Genomic copy number anomalies and their association to lncRNAs

The GISTIC 2.0 tool^51^ was used to define CNA extracted from the TCGA-LIHC dataset with a cutoff value of gain /loss threshold > 0.1 and Q < 0.25 (When using GISTIC2 to the detect significantly gain or loss genomic regions in a group of samples, the integration of all results of gistic can be obtained, including gain and loss regions, and the samples of gain or loss in each region and the Q value in the peak region are acquired). Copy numbers ≥ 1 or ≤ −1 were considered gain and loss, respectively. The variant frequency of lncRNAs in samples was calculated, and the copy number gain and loss distribution of lncRNA in the genome were analyzed using the Rcircos tool^52^. Samples with lncRNA expression profiles were chosen, and Pearson correlation coefficients between CNA and lncRNA expression were analyzed. In addition, the differences of the expression of lncRNA with CNA frequency > 75% between normal, copy loss, and gain samples were compared using Kruskal–Wallis test.

### Screening lncRNA prognostic markers with CNA and construction of lncRNA signature

First, the lncRNAs that met the following criteria were considered as candidate lncRNAs: CNA frequency > 5%, a significant positive correlation between CNA and expression (correlation coefficient > 0.3 and P < 0.05), and DE between different subtypes. Univariate Cox regression analysis was then performed to identify prognostic-related lncRNAs using the Survminer package (P < 0.05)^53^. In addition, the samples in the TCGA database were categorized into training set (n = 234) and testing set (n = 101) based on 7:3, and the whole sample dataset was used as the validation set (n = 335). In the training set, the LASSO Cox regression analysis was performed using the glmnet package^54^, and a 20-fold cross-validation was utilized to build the lncRNA signature. The risk score was analyzed using the following formula: Risk score = β_lncRNA1_ × expr_lncRNA1_ + β_lncRNA2_ × expr_lncRNA2_ + … + β_lncRNAn_ × expr_lncRNAn_ (β represents the regression coefficient of lncRNAs, and expr represents the lncRNA expression level). The samples were then categorized into High_ and Low_risk groups based on the median risk score. Kaplan–Meier survival curve analysis was then conducted. In addition, the model was validated in the testing and validation sets. To verify the prognostic performance of the lncRNA signature, receiver operating characteristic (ROC) analysis was carried out in three sets using the survivalROC package^55^.

### Development of the nomogram

Nomogram is a method to display the results of the signature intuitively and effectively, and is conveniently applied in the prediction of the outcome. It uses the length of the line to represent the different variables, thereby exhibiting the effect of different variable values on the outcome. To test whether the Riskscore model was an independent prognostic factor, univariate and multivariate Cox regression analyses were carried out on RiskGroup and clinical characteristics, including AGE, AJCC_PATHOLOGIC_TUMOR_STAGE, and GRADE, and the characteristics with P < 0.05, were used to build a nomogram. The nomogram was validated by assessing the discrimination and calibration. To be clear, the calibration curve of the nomogram was plotted to observe the nomogram prediction probabilities against the observed rates.

### Clinical characteristics

The distribution of each clinical characteristic in High_ and Low_risk groups was statistically explored, and the differences were compared using the Chi-square test. In addition, the distribution of subtype samples between the High_ and Low_risk groups was evaluated.

### Tumor immune microenvironment

Stromal, immune, estimated scores, and tumor purity were evaluated using the ESTIMATE algorithm^56^. In addition, CIBERSORT^57^ was employed to evaluate the fractions of 22 tumor-infiltrating immune cells, and the differences in stromal score, immune score, estimate score, tumor purity, and fractions of 22 tumor-infiltrating immune cells were analyzed using the Wilcox test.

### Drug sensitivity prediction

The Genomics of Drug Sensitivity in Cancer (GDSC) database^58^ was utilized to assess the sensitivity of patients in High_ and Low_risk groups to chemotherapy drugs. The IC50 of 138 drugs was calculated using the pRRophetic algorithm^59^ in R, and the differences were compared using the t-test.

### Identification of enriched lncRNA modules by WGCNA

The WGCNA package^28^ was employed to build a scale-free co-expression network based on the combined expression profiles of DEmRNAs and lncRNAs in the prognostic model, and the highly covarying gene set modules were identified. The mRNA in the same module serves as a potential lncRNA target gene. Enrichment analysis was conducted on the mRNA in the modules using clusterprofiler^60^, with the parameters of pAdjustMethod = “BH” and P < 0.05.

## Supplementary Information

Supplementary Figures.

Supplementary Table 1.

Supplementary Table 2.

## Data Availability

(1) The gene expression RNA-seq (log2(fpkm + 1)) data of GDC TCGA LIHC were downloaded from the UCSC Xene platform (https://xenabrowser.net/). (2) Clinical and survival information in the TCGA database were obtained from the cBioportal website (http://cbioportal.org).

## Abbreviations

lncRNA: Long non-coding RNAs
DE: Differential expression
mRNAs: Messenger RNAs
TCGA: The Cancer Genome Atlas
OS: Overall survival
LASSO: Least absolute shrinkage and selection operator
WGCNA: Weighted correlation network analysis
KEGG: Kyoto Encyclopedia of Genes and Genomes
