# Supplementary material for: Four-copy number alteration (CNA)-related lncRNA prognostic signature for liver cancer
Source: Sci Rep. 2022 Aug 22;12:14261. doi: 10.1038/s41598-022-17927-0 (PMC9395537; doi:10.1038/s41598-022-17927-0)

**Sup Fig. 1. Identification of differentially expressed (DE) mRNAs and DElncRNAs in two subtypes.** Volcano plot of (A) DEmRNA and (B) DElncRNA.

**
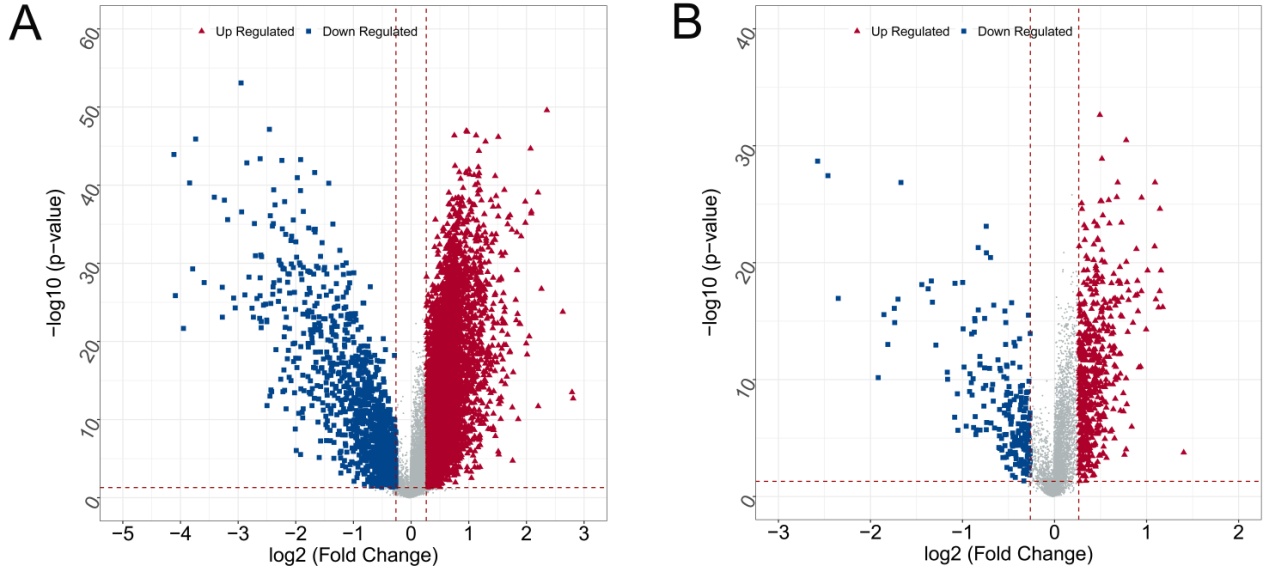
**

**Sup Fig. 2. Expression of lncRNAs with CNA frequency > 75% in the samples with copy gain and normal copies.** Green column indicates loss, yellow column indicates diploid, and red column indicates gain.

**
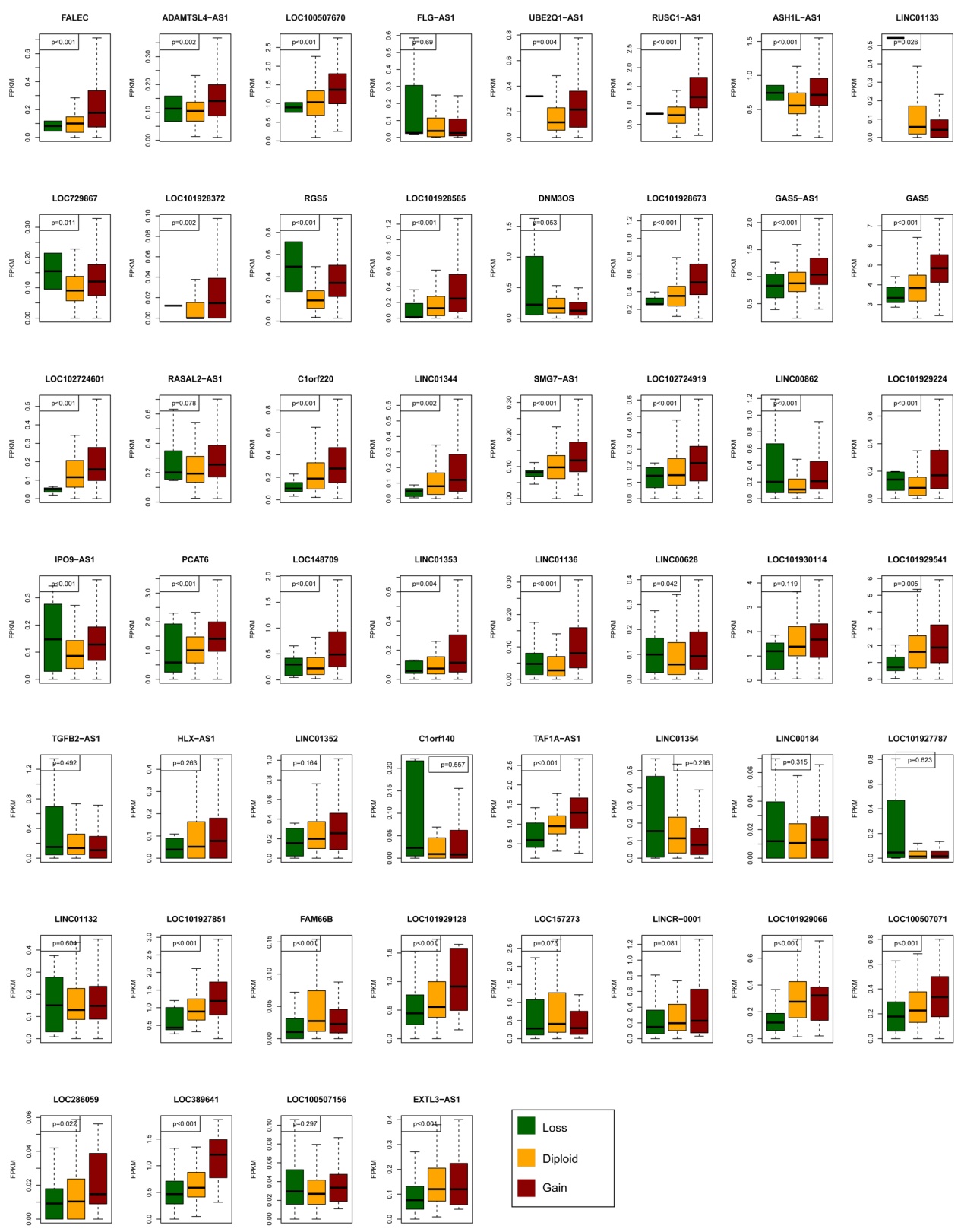
**

**Sup Fig. 3. Difference in distribution of subtype samples in the High_ and Low_risk groups.**

**
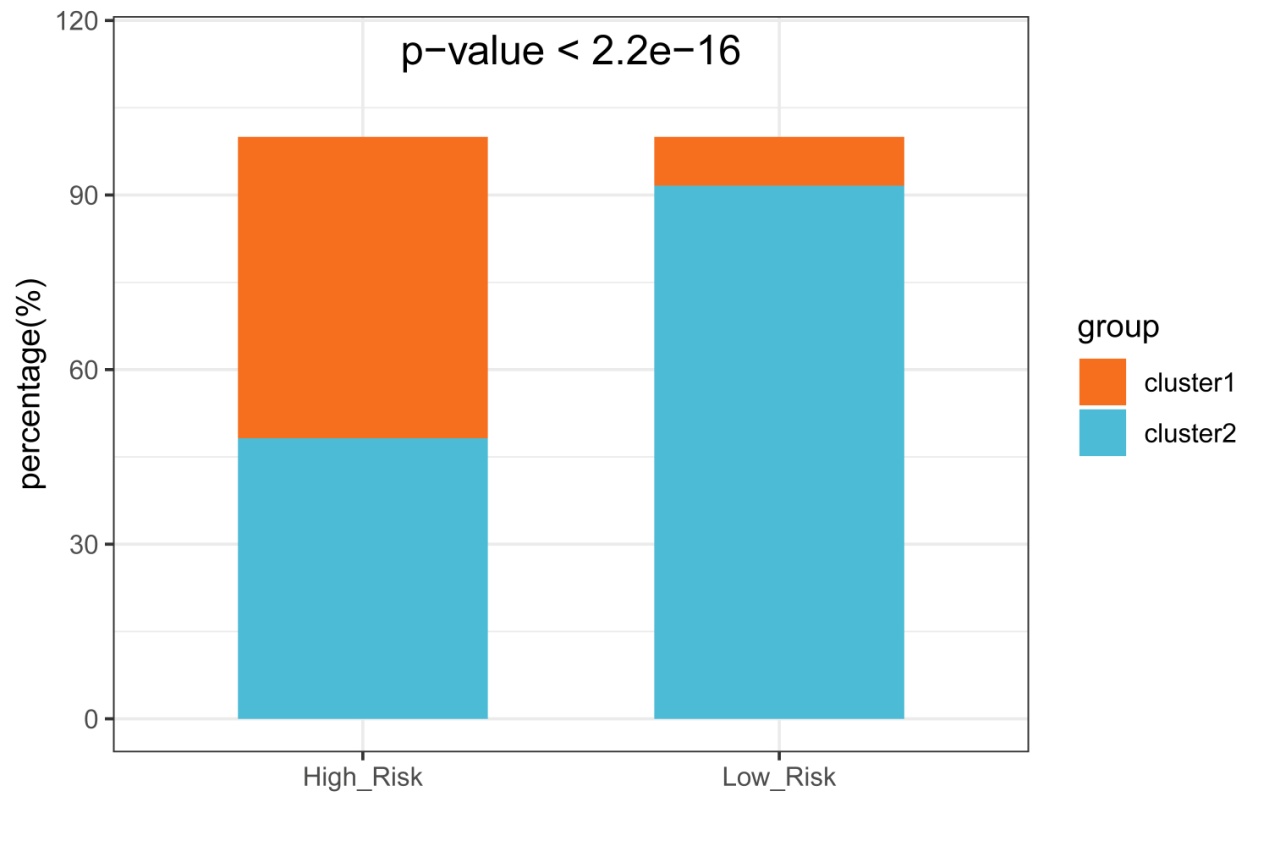
**

**Sup Fig. 4. Drug sensitivity prediction.** Yellow column indicates the Low_risk group, while blue column indicates the High_risk group.


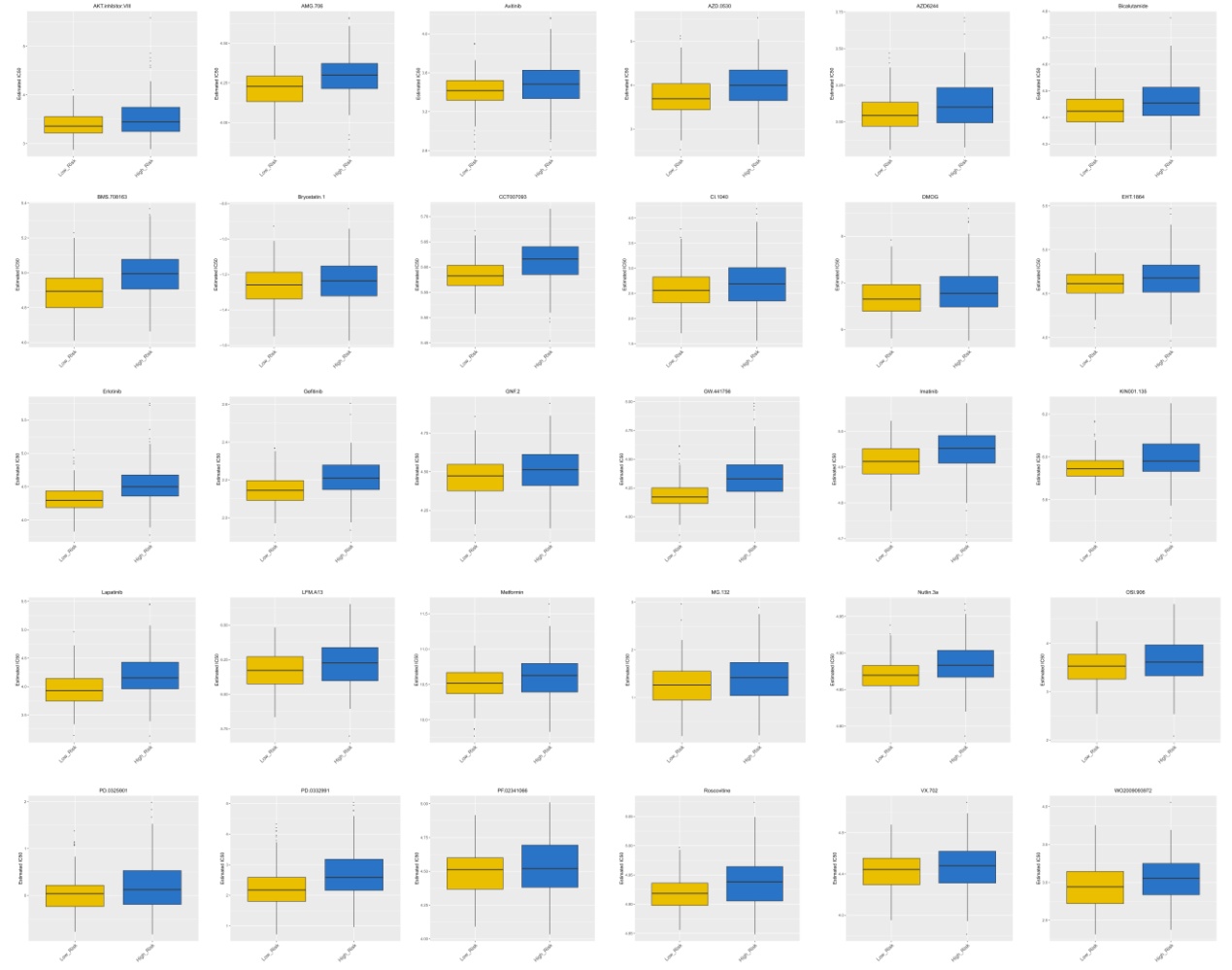

Supplement: Supplementary file 1 — Supplementary Figures. [file 41598_2022_17927_MOESM1_ESM.docx]
