# Supplementary material for: Four-copy number alteration (CNA)-related lncRNA prognostic signature for liver cancer
Source: Sci Rep. 2022 Aug 22;12:14261. doi: 10.1038/s41598-022-17927-0 (PMC9395537; doi:10.1038/s41598-022-17927-0)
Supplement: Supplementary file 2 — Supplementary Table 1. [file 41598_2022_17927_MOESM2_ESM.docx]

Supplementary Table 1: Total 1,238 lncRNAs had significant difference on CNA between cluster1 and cluster2.

| lncRNA | p.value |
| --- | --- |
| DHRS4-AS1 | 1.51E-14 |
| PRMT5-AS1 | 2.11E-14 |
| LOC101926933 | 2.97E-14 |
| LINC00641 | 4.15E-14 |
| LOC101929718 | 5.87E-14 |
| LOC100506071 | 1.74E-13 |
| LOC101927124 | 1.75E-13 |
| LINC00639 | 1.75E-13 |
| LOC102724814 | 2.12E-13 |
| LOC100288846 | 4.26E-13 |
| SLC25A21-AS1 | 7.65E-13 |
| LOC101928725 | 2.12E-12 |
| RORA-AS1 | 5.87E-12 |
| LIPC-AS1 | 1.53E-11 |
| USP3-AS1 | 2.81E-11 |
| LINC01588 | 2.81E-11 |
| GOLGA8M | 3.22E-11 |
| LOC100130111 | 3.22E-11 |
| LOC101928988 | 3.71E-11 |
| LINC00540 | 4.88E-11 |
| LOC101927418 | 5.50E-11 |
| EWSAT1 | 5.70E-11 |
| LOC145694 | 5.70E-11 |
| PCAT29 | 5.70E-11 |
| FRMD6-AS1 | 6.04E-11 |
| LINC00346 | 7.70E-11 |
| ARHGEF7-AS2 | 7.70E-11 |
| LOC283683 | 8.84E-11 |
| LINC00640 | 1.24E-10 |
| COL4A2-AS1 | 1.25E-10 |
| LINC00926 | 1.43E-10 |
| CLYBL-AS2 | 1.49E-10 |
| LINC01169 | 1.71E-10 |
| LINC00565 | 1.88E-10 |
| LOC100131315 | 2.31E-10 |
| GAS6-AS2 | 2.59E-10 |
| LOC105370333 | 2.60E-10 |
| IQCH-AS1 | 2.74E-10 |
| FGF14-AS2 | 3.24E-10 |
| C15orf54 | 3.24E-10 |
| LINC00426 | 3.55E-10 |
| LINC01058 | 3.55E-10 |
| OIP5-AS1 | 3.85E-10 |
| LINC00399 | 4.08E-10 |
| NALCN-AS1 | 4.28E-10 |
| LINC00327 | 5.14E-10 |
| CASC22 | 6.79E-10 |
| LINC00449 | 6.92E-10 |
| SRP14-AS1 | 7.08E-10 |
| UBAC2-AS1 | 7.99E-10 |
| LOC101927272 | 1.18E-09 |
| NPTN-IT1 | 1.60E-09 |
| SPATA13 | 1.91E-09 |
| LINC00365 | 2.04E-09 |
| LINC00543 | 2.06E-09 |
| MIR17HG | 2.11E-09 |
| LINC00593 | 2.16E-09 |
| LOC105371267 | 2.45E-09 |
| ACTN1-AS1 | 2.69E-09 |
| RAD51-AS1 | 2.78E-09 |
| DOCK9-AS1 | 3.27E-09 |
| LINC00398 | 3.30E-09 |
| PLA2G4E-AS1 | 4.12E-09 |
| LMF1-AS1 | 4.32E-09 |
| LINC01146 | 4.45E-09 |
| FOXN3-AS1 | 4.45E-09 |
| LOXL1-AS1 | 5.13E-09 |
| LOC283731 | 5.13E-09 |
| LMF1 | 5.85E-09 |
| LCMT1-AS2 | 6.03E-09 |
| LOC105370941 | 6.15E-09 |
| KTN1-AS1 | 6.70E-09 |
| HAND2-AS1 | 7.26E-09 |
| HEXA-AS1 | 8.12E-09 |
| MIR4500HG | 8.31E-09 |
| LOC101928414 | 9.15E-09 |
| LOC105371083 | 9.60E-09 |
| LOC102724153 | 9.93E-09 |
| LINC00239 | 9.97E-09 |
| WASIR2 | 1.07E-08 |
| LOC101930370 | 1.12E-08 |
| INAFM2 | 1.13E-08 |
| C14orf132 | 1.18E-08 |
| FLJ22447 | 1.20E-08 |
| LINC01269 | 1.23E-08 |
| ADAM20P1 | 1.38E-08 |
| STARD13-AS | 1.47E-08 |
| LINC00677 | 1.65E-08 |
| LOC102723809 | 1.71E-08 |
| LOC101928858 | 1.83E-08 |
| LINC01550 | 2.00E-08 |
| LOC283856 | 2.20E-08 |
| LINC00638 | 2.24E-08 |
| HIF1A-AS1 | 2.52E-08 |
| DIO3OS | 2.52E-08 |
| TCL6 | 2.62E-08 |
| ADPGK-AS1 | 2.67E-08 |
| LINC00605 | 2.67E-08 |
| LINC00348 | 2.83E-08 |
| LOC101929439 | 2.99E-08 |
| LOC101928002 | 3.34E-08 |
| RMST | 3.51E-08 |
| N4BP2L2-IT2 | 3.87E-08 |
| PSMA3-AS1 | 4.11E-08 |
| LOC100507437 | 4.12E-08 |
| LMO7-AS1 | 4.25E-08 |
| LINC01337 | 4.51E-08 |
| LOC100506321 | 4.56E-08 |
| LOC105371184 | 4.67E-08 |
| DICER1-AS1 | 4.68E-08 |
| LINC01465 | 4.75E-08 |
| LINC00571 | 4.97E-08 |
| SSTR5-AS1 | 5.45E-08 |
| ENTPD1-AS1 | 5.49E-08 |
| LOC102723385 | 6.23E-08 |
| LINC00637 | 6.46E-08 |
| MEG3 | 6.88E-08 |
| LOC101927480 | 6.99E-08 |
| SNHG9 | 7.34E-08 |
| MGC27382 | 8.71E-08 |
| AP4B1-AS1 | 8.76E-08 |
| LOC106660606 | 1.02E-07 |
| MEG9 | 1.04E-07 |
| UBL7-AS1 | 1.04E-07 |
| MGC32805 | 1.08E-07 |
| FAM30A | 1.08E-07 |
| LINC00221 | 1.08E-07 |
| SNHG21 | 1.20E-07 |
| MIR9-3HG | 1.27E-07 |
| SRD5A3-AS1 | 1.28E-07 |
| TMPO-AS1 | 1.32E-07 |
| HIPK1-AS1 | 1.48E-07 |
| INTS6-AS1 | 1.52E-07 |
| LOC100505915 | 1.58E-07 |
| LOC102723692 | 1.58E-07 |
| PKD1P6-NPIPP1 | 1.58E-07 |
| MIR3681HG | 1.61E-07 |
| LOC105369971 | 1.74E-07 |
| LOC729970 | 1.75E-07 |
| HHIP-AS1 | 1.78E-07 |
| RNF144A-AS1 | 1.79E-07 |
| LINC01481 | 1.86E-07 |
| LINC00548 | 1.86E-07 |
| LINC00598 | 1.86E-07 |
| CTD-2201I18.1 | 1.91E-07 |
| RASGRF2-AS1 | 1.91E-07 |
| CKMT2-AS1 | 1.91E-07 |
| BCDIN3D-AS1 | 1.93E-07 |
| RNASEH1-AS1 | 2.03E-07 |
| LINC01140 | 2.09E-07 |
| OLMALINC | 2.16E-07 |
| LOC339666 | 2.17E-07 |
| LINC01091 | 2.17E-07 |
| NEXN-AS1 | 2.21E-07 |
| FLJ31104 | 2.33E-07 |
| SNHG19 | 2.33E-07 |
| LOC101929140 | 2.46E-07 |
| LOC101927560 | 2.52E-07 |
| LOC101926964 | 2.56E-07 |
| RGMB-AS1 | 2.56E-07 |
| LINC00960 | 2.60E-07 |
| LINC00989 | 2.64E-07 |
| SMIM2-AS1 | 2.65E-07 |
| LOC105371049 | 2.74E-07 |
| CRTC3-AS1 | 2.83E-07 |
| LOC105377267 | 2.90E-07 |
| IL21R-AS1 | 3.14E-07 |
| SLC25A30-AS1 | 3.34E-07 |
| CPB2-AS1 | 3.35E-07 |
| PYCARD-AS1 | 3.41E-07 |
| LOC100507487 | 3.60E-07 |
| VWA8-AS1 | 3.68E-07 |
| TRHDE-AS1 | 3.69E-07 |
| PKN2-AS1 | 3.71E-07 |
| SCAMP1-AS1 | 3.78E-07 |
| LOC101928737 | 3.93E-07 |
| LOC101929710 | 4.03E-07 |
| ID2-AS1 | 4.08E-07 |
| RBM26-AS1 | 4.17E-07 |
| SLC16A1-AS1 | 4.19E-07 |
| LOC100996251 | 4.19E-07 |
| ALKBH3-AS1 | 4.23E-07 |
| LINC01572 | 4.26E-07 |
| LOC105378853 | 4.33E-07 |
| PCED1B-AS1 | 4.43E-07 |
| TAT-AS1 | 4.47E-07 |
| NFIA-AS2 | 4.52E-07 |
| PINK1-AS | 4.59E-07 |
| LINC00339 | 4.59E-07 |
| LOC729652 | 4.74E-07 |
| SRRM2-AS1 | 4.74E-07 |
| ZNF436-AS1 | 4.75E-07 |
| LOC257396 | 4.77E-07 |
| PROSER2-AS1 | 4.85E-07 |
| MIR762HG | 4.85E-07 |
| VAC14-AS1 | 4.86E-07 |
| PART1 | 4.90E-07 |
| SMAD5-AS1 | 4.90E-07 |
| WWC2-AS2 | 5.05E-07 |
| TSC22D1-AS1 | 5.09E-07 |
| IFNG-AS1 | 5.11E-07 |
| LOC101929147 | 5.15E-07 |
| F11-AS1 | 5.17E-07 |
| LOC100507377 | 5.20E-07 |
| LOC101928659 | 5.22E-07 |
| KCNIP2-AS1 | 5.28E-07 |
| DPYD-AS1 | 5.54E-07 |
| LINC01554 | 5.55E-07 |
| LOC643201 | 5.55E-07 |
| FLJ21408 | 5.57E-07 |
| FBXL19-AS1 | 5.67E-07 |
| LINC00304 | 5.79E-07 |
| LOC101927793 | 5.79E-07 |
| SNAI3-AS1 | 5.79E-07 |
| DLEU2 | 5.80E-07 |
| DLEU7-AS1 | 5.80E-07 |
| DBET | 5.95E-07 |
| LINC00870 | 5.97E-07 |
| LOC105377623 | 6.10E-07 |
| LINC01356 | 6.44E-07 |
| FENDRR | 6.48E-07 |
| ATP1A1-AS1 | 6.66E-07 |
| LINC00622 | 6.70E-07 |
| LOC101927895 | 6.77E-07 |
| LINC00487 | 6.92E-07 |
| LINC00563 | 7.19E-07 |
| C2orf48 | 7.23E-07 |
| LINC00877 | 7.25E-07 |
| LOC101927237 | 7.35E-07 |
| CRAT37 | 7.44E-07 |
| LOC101928731 | 7.54E-07 |
| LINC00562 | 8.42E-07 |
| MED4-AS1 | 8.42E-07 |
| MYCBP2-AS1 | 8.48E-07 |
| LOC283194 | 8.52E-07 |
| LOC105378828 | 8.73E-07 |
| PRC1-AS1 | 8.85E-07 |
| LINC01184 | 8.85E-07 |
| TUSC8 | 9.07E-07 |
| LOC101929657 | 9.16E-07 |
| LOC100506474 | 9.21E-07 |
| UBA6-AS1 | 9.35E-07 |
| LINC01359 | 9.56E-07 |
| CROCCP2 | 9.63E-07 |
| LOC105376805 | 9.63E-07 |
| LOC100507250 | 9.65E-07 |
| LOC101929162 | 1.02E-06 |
| TMEM220-AS1 | 1.03E-06 |
| LOC100506083 | 1.03E-06 |
| FLJ13224 | 1.07E-06 |
| ATP2A1-AS1 | 1.07E-06 |
| FAM212B-AS1 | 1.08E-06 |
| UBXN10-AS1 | 1.09E-06 |
| ZSWIM8-AS1 | 1.10E-06 |
| LOC101929099 | 1.10E-06 |
| SPRY4-IT1 | 1.12E-06 |
| SNHG8 | 1.15E-06 |
| LOC101929741 | 1.15E-06 |
| LOC400553 | 1.15E-06 |
| LINC00298 | 1.17E-06 |
| LINC00299 | 1.17E-06 |
| HAO2-IT1 | 1.26E-06 |
| LINC01336 | 1.27E-06 |
| HTR2A-AS1 | 1.28E-06 |
| LOC731157 | 1.28E-06 |
| TMEM51-AS1 | 1.31E-06 |
| GS1-124K5.4 | 1.32E-06 |
| GS1-124K5.11 | 1.32E-06 |
| LOC101928098 | 1.32E-06 |
| NR2F1-AS1 | 1.35E-06 |
| VPS9D1-AS1 | 1.37E-06 |
| PLCE1-AS1 | 1.37E-06 |
| DKFZp779M0652 | 1.37E-06 |
| SH3PXD2A-AS1 | 1.40E-06 |
| LUCAT1 | 1.43E-06 |
| ARRDC3-AS1 | 1.43E-06 |
| LOC105377590 | 1.46E-06 |
| C17orf100 | 1.49E-06 |
| LOC101928837 | 1.52E-06 |
| LOC101928035 | 1.61E-06 |
| LOC283335 | 1.63E-06 |
| TH2LCRR | 1.65E-06 |
| LINC00941 | 1.73E-06 |
| RNF219-AS1 | 1.79E-06 |
| DNAJC9-AS1 | 1.79E-06 |
| BEAN1-AS1 | 1.80E-06 |
| KCTD21-AS1 | 1.83E-06 |
| LINC00923 | 1.84E-06 |
| LOC101928530 | 1.85E-06 |
| LOC102724163 | 1.91E-06 |
| LOC101928812 | 1.97E-06 |
| LINC00865 | 1.99E-06 |
| DUXAP8 | 2.04E-06 |
| TPTEP1 | 2.04E-06 |
| FLJ37453 | 2.05E-06 |
| C10orf95 | 2.05E-06 |
| RPARP-AS1 | 2.05E-06 |
| LOC100506801 | 2.09E-06 |
| LOC105369911 | 2.13E-06 |
| PLBD1-AS1 | 2.14E-06 |
| LOC102724450 | 2.15E-06 |
| CASC18 | 2.18E-06 |
| THAP9-AS1 | 2.29E-06 |
| LINC01135 | 2.29E-06 |
| SEMA6A-AS1 | 2.40E-06 |
| C5orf64 | 2.41E-06 |
| LOC101928118 | 2.41E-06 |
| OVCH1-AS1 | 2.41E-06 |
| STARD4-AS1 | 2.42E-06 |
| TMEM72-AS1 | 2.65E-06 |
| FAM66C | 2.67E-06 |
| LINC00937 | 2.67E-06 |
| WDFY3-AS2 | 2.73E-06 |
| SVIL-AS1 | 2.83E-06 |
| LOC105376382 | 2.83E-06 |
| USP46-AS1 | 2.90E-06 |
| DANCR | 2.90E-06 |
| TMEM161B-AS1 | 2.92E-06 |
| MEF2C-AS1 | 2.92E-06 |
| LOC284454 | 3.00E-06 |
| ATE1-AS1 | 3.00E-06 |
| GNG12-AS1 | 3.00E-06 |
| CECR7 | 3.03E-06 |
| DDX11-AS1 | 3.06E-06 |
| LOC101928053 | 3.06E-06 |
| ZBED5-AS1 | 3.06E-06 |
| LOC101928266 | 3.07E-06 |
| LINC00528 | 3.18E-06 |
| LOC102724404 | 3.20E-06 |
| LINC00954 | 3.22E-06 |
| LOC100996842 | 3.26E-06 |
| CCDC192 | 3.29E-06 |
| LOC100506606 | 3.29E-06 |
| IGFBP7-AS1 | 3.29E-06 |
| MAFTRR | 3.34E-06 |
| LINC01229 | 3.34E-06 |
| SLC7A11-AS1 | 3.37E-06 |
| LINC01023 | 3.50E-06 |
| SOCS2-AS1 | 3.50E-06 |
| LOC101928865 | 3.54E-06 |
| LOC100128770 | 3.56E-06 |
| GLIS2-AS1 | 3.56E-06 |
| LINC00924 | 3.57E-06 |
| PARD3-AS1 | 3.66E-06 |
| LINC00485 | 3.75E-06 |
| ZEB1-AS1 | 3.84E-06 |
| MATN1-AS1 | 3.87E-06 |
| LINC00570 | 4.11E-06 |
| LOC285593 | 4.16E-06 |
| ZRANB2-AS2 | 4.19E-06 |
| LINC01484 | 4.21E-06 |
| PPP3CB-AS1 | 4.28E-06 |
| LOC100506551 | 4.35E-06 |
| SNAP25-AS1 | 4.38E-06 |
| LOC730183 | 4.48E-06 |
| LOC101929295 | 4.55E-06 |
| LOC100506125 | 4.61E-06 |
| LOC339260 | 4.78E-06 |
| LOC100507144 | 4.83E-06 |
| ALOX12-AS1 | 4.84E-06 |
| MIR497HG | 4.84E-06 |
| FLJ12825 | 4.85E-06 |
| LINC00939 | 5.08E-06 |
| ZNF337-AS1 | 5.17E-06 |
| FAM182A | 5.17E-06 |
| LOC101928622 | 5.19E-06 |
| LOC399715 | 5.31E-06 |
| C10orf111 | 5.34E-06 |
| TMEM9B-AS1 | 5.37E-06 |
| LINC01559 | 5.53E-06 |
| LINC01428 | 5.53E-06 |
| LINC00294 | 5.63E-06 |
| TNKS2-AS1 | 5.68E-06 |
| C22orf24 | 5.69E-06 |
| SEC24B-AS1 | 5.70E-06 |
| RPL34-AS1 | 5.70E-06 |
| HS1BP3-IT1 | 5.72E-06 |
| COX10-AS1 | 5.78E-06 |
| LINC00921 | 5.89E-06 |
| LOC101928100 | 5.90E-06 |
| SENCR | 5.90E-06 |
| ALG1L9P | 5.91E-06 |
| CCDC144NL-AS1 | 6.00E-06 |
| RAB30-AS1 | 6.09E-06 |
| LINC01003 | 6.23E-06 |
| LOC102724064 | 6.26E-06 |
| LOC400464 | 6.32E-06 |
| CASC2 | 6.32E-06 |
| MYCNOS | 6.37E-06 |
| UNC5B-AS1 | 6.37E-06 |
| SCARNA9 | 6.48E-06 |
| LOC105376736 | 6.72E-06 |
| LINC00311 | 6.77E-06 |
| SPATA41 | 6.79E-06 |
| LINC01569 | 6.82E-06 |
| MRVI1-AS1 | 6.94E-06 |
| NAV2-AS5 | 7.01E-06 |
| NAV2-AS4 | 7.01E-06 |
| SLC8A1-AS1 | 7.12E-06 |
| LOC100505716 | 7.12E-06 |
| LOC106699570 | 7.20E-06 |
| FLJ31356 | 7.28E-06 |
| LOC100506258 | 7.46E-06 |
| LINC00930 | 7.55E-06 |
| SCOC-AS1 | 7.63E-06 |
| ATP6V0E2-AS1 | 7.83E-06 |
| LOC102724927 | 7.90E-06 |
| LINC01197 | 8.05E-06 |
| NR2F2-AS1 | 8.05E-06 |
| LOC100996419 | 8.05E-06 |
| LOC221122 | 8.11E-06 |
| WEE2-AS1 | 8.11E-06 |
| HOXC-AS2 | 8.63E-06 |
| C11orf72 | 8.89E-06 |
| LOC100287944 | 8.90E-06 |
| LINC01376 | 8.92E-06 |
| LOC102724050 | 8.95E-06 |
| ENTPD3-AS1 | 9.00E-06 |
| LOC101929719 | 9.07E-06 |
| LOC101929595 | 9.08E-06 |
| GRPEL2-AS1 | 9.15E-06 |
| ITGA9-AS1 | 9.16E-06 |
| MGC12916 | 9.23E-06 |
| LOC101927051 | 9.29E-06 |
| LINC01137 | 9.52E-06 |
| HNF1A-AS1 | 9.52E-06 |
| WDR11-AS1 | 9.73E-06 |
| CCND2-AS1 | 9.91E-06 |
| LINC00839 | 1.01E-05 |
| LOC101928069 | 1.04E-05 |
| LINC00173 | 1.07E-05 |
| BMS1P4 | 1.09E-05 |
| LOC105372273 | 1.10E-05 |
| LOC101928445 | 1.12E-05 |
| BDNF-AS | 1.16E-05 |
| JAKMIP2-AS1 | 1.16E-05 |
| LOC100507373 | 1.20E-05 |
| LINC00619 | 1.24E-05 |
| LINC00840 | 1.24E-05 |
| C11orf97 | 1.24E-05 |
| DENND5B-AS1 | 1.30E-05 |
| LINC00847 | 1.31E-05 |
| LOC644656 | 1.31E-05 |
| LINC01234 | 1.32E-05 |
| LOC105371592 | 1.33E-05 |
| C7orf13 | 1.35E-05 |
| LOC100506691 | 1.35E-05 |
| LINC00324 | 1.37E-05 |
| LOC100288123 | 1.38E-05 |
| LOC285889 | 1.40E-05 |
| LINC01006 | 1.40E-05 |
| LINC00244 | 1.40E-05 |
| LOC100268168 | 1.40E-05 |
| LOC105378663 | 1.40E-05 |
| LINC01287 | 1.44E-05 |
| LOC100653233 | 1.44E-05 |
| KIZ-AS1 | 1.47E-05 |
| CD27-AS1 | 1.47E-05 |
| LOC105369486 | 1.47E-05 |
| ZNF503-AS1 | 1.51E-05 |
| LOC101929464 | 1.51E-05 |
| ELFN2 | 1.53E-05 |
| C3orf35 | 1.56E-05 |
| NRAV | 1.60E-05 |
| LOC653160 | 1.64E-05 |
| FOXP1-AS1 | 1.64E-05 |
| GTF3C2-AS1 | 1.68E-05 |
| LOC102724908 | 1.70E-05 |
| ENO1-AS1 | 1.73E-05 |
| LOC440028 | 1.73E-05 |
| LOC648987 | 1.77E-05 |
| LOC153684 | 1.77E-05 |
| LOC100506585 | 1.77E-05 |
| LOC101928728 | 1.78E-05 |
| ST8SIA6-AS1 | 1.83E-05 |
| STAM-AS1 | 1.83E-05 |
| LINC00940 | 1.84E-05 |
| EIF1B-AS1 | 1.85E-05 |
| LOC728730 | 1.85E-05 |
| LINC00689 | 1.87E-05 |
| LINC00654 | 1.90E-05 |
| LOC105379143 | 1.93E-05 |
| OR51B5 | 1.93E-05 |
| LOC101927751 | 1.94E-05 |
| DPP9-AS1 | 1.94E-05 |
| DKFZP434K028 | 1.95E-05 |
| LOC105376671 | 1.96E-05 |
| FAM87B | 1.98E-05 |
| LINC00115 | 1.98E-05 |
| LINC00982 | 1.98E-05 |
| LINC01128 | 1.98E-05 |
| LOC100130417 | 1.98E-05 |
| LOC100996583 | 1.98E-05 |
| LOC148413 | 1.98E-05 |
| FLJ20021 | 1.98E-05 |
| EMX2OS | 1.99E-05 |
| MKLN1-AS | 2.01E-05 |
| LOC101927322 | 2.01E-05 |
| GHET1 | 2.04E-05 |
| LOC101929427 | 2.08E-05 |
| MRGPRF-AS1 | 2.08E-05 |
| LOC101927809 | 2.12E-05 |
| LEF1-AS1 | 2.14E-05 |
| LOC101928887 | 2.15E-05 |
| OGFRP1 | 2.16E-05 |
| LOC101928000 | 2.17E-05 |
| LOC100130950 | 2.17E-05 |
| CACTIN-AS1 | 2.20E-05 |
| LOC283140 | 2.21E-05 |
| LOC105369332 | 2.21E-05 |
| SNHG1 | 2.21E-05 |
| LOC105379194 | 2.24E-05 |
| FAM13A-AS1 | 2.26E-05 |
| KLF3-AS1 | 2.26E-05 |
| SNHG12 | 2.27E-05 |
| UBOX5-AS1 | 2.32E-05 |
| LINC01089 | 2.35E-05 |
| SFTA1P | 2.36E-05 |
| LOC102724094 | 2.37E-05 |
| LOC101927762 | 2.42E-05 |
| MIR34AHG | 2.43E-05 |
| DNAJC27-AS1 | 2.45E-05 |
| DSCAS | 2.46E-05 |
| URB1-AS1 | 2.47E-05 |
| LOC100130705 | 2.48E-05 |
| ACVR2B-AS1 | 2.49E-05 |
| TINCR | 2.50E-05 |
| LOC283038 | 2.52E-05 |
| FLJ37035 | 2.52E-05 |
| EDRF1-AS1 | 2.52E-05 |
| LINC01537 | 2.65E-05 |
| LOC100129138 | 2.69E-05 |
| LINC01121 | 2.75E-05 |
| SMCR2 | 2.78E-05 |
| CTB-178M22.2 | 2.82E-05 |
| FAM222A-AS1 | 2.85E-05 |
| LOC100128568 | 2.87E-05 |
| SBF2-AS1 | 2.88E-05 |
| TP73-AS1 | 2.90E-05 |
| LINC01134 | 2.90E-05 |
| VIM-AS1 | 2.92E-05 |
| SSBP3-AS1 | 2.92E-05 |
| LINC01252 | 2.95E-05 |
| LINC01191 | 2.95E-05 |
| LOC100505549 | 2.97E-05 |
| LOC400684 | 3.00E-05 |
| C10orf25 | 3.00E-05 |
| ACTA2-AS1 | 3.02E-05 |
| LOC105377348 | 3.03E-05 |
| NDUFB2-AS1 | 3.07E-05 |
| LOC101927257 | 3.09E-05 |
| LOC101928844 | 3.10E-05 |
| MIAT | 3.18E-05 |
| SNHG11 | 3.32E-05 |
| SNHG3 | 3.35E-05 |
| LOC101928111 | 3.37E-05 |
| LINC00863 | 3.39E-05 |
| NUTM2A-AS1 | 3.39E-05 |
| PXN-AS1 | 3.43E-05 |
| LINC00853 | 3.54E-05 |
| PIK3IP1-AS1 | 3.55E-05 |
| FEZF1-AS1 | 3.56E-05 |
| LOC101929516 | 3.59E-05 |
| LOC100506142 | 3.62E-05 |
| MKNK1-AS1 | 3.66E-05 |
| LHFPL3-AS2 | 3.72E-05 |
| C1RL-AS1 | 3.78E-05 |
| LRP4-AS1 | 3.80E-05 |
| LOC105369340 | 3.80E-05 |
| LOC100996455 | 3.80E-05 |
| SMIM10L1 | 3.81E-05 |
| MALAT1 | 3.90E-05 |
| NEAT1 | 3.90E-05 |
| OVOL1-AS1 | 3.90E-05 |
| ZNF32-AS1 | 3.97E-05 |
| ZNF32-AS2 | 3.97E-05 |
| LRRC75A-AS1 | 3.98E-05 |
| LOC102724301 | 4.07E-05 |
| LINC01532 | 4.09E-05 |
| AGBL5-AS1 | 4.10E-05 |
| LOC100128531 | 4.17E-05 |
| LOC105372493 | 4.20E-05 |
| SDCBP2-AS1 | 4.20E-05 |
| TAPT1-AS1 | 4.24E-05 |
| LOC101929577 | 4.27E-05 |
| LINC00996 | 4.32E-05 |
| PCAT18 | 4.32E-05 |
| ZNF503-AS2 | 4.43E-05 |
| LINC01431 | 4.50E-05 |
| LOC101929340 | 4.51E-05 |
| LOC100506368 | 4.63E-05 |
| MIR100HG | 4.63E-05 |
| LOC102724323 | 4.67E-05 |
| LOC101929549 | 4.68E-05 |
| LOC105371795 | 4.73E-05 |
| ADAMTS9-AS2 | 4.75E-05 |
| JMJD1C-AS1 | 4.76E-05 |
| LOC284788 | 4.82E-05 |
| LOC102725254 | 4.89E-05 |
| SERHL | 4.94E-05 |
| TTC28-AS1 | 4.98E-05 |
| LIPE-AS1 | 5.01E-05 |
| LOC100128386 | 5.01E-05 |
| WDR86-AS1 | 5.01E-05 |
| BCYRN1 | 5.05E-05 |
| SLC2A1-AS1 | 5.06E-05 |
| LOC100049716 | 5.07E-05 |
| LOC102723544 | 5.07E-05 |
| LOC574538 | 5.07E-05 |
| ZNF561-AS1 | 5.10E-05 |
| LOC102546294 | 5.17E-05 |
| LINC00612 | 5.21E-05 |
| LOC101929095 | 5.28E-05 |
| ST7-AS1 | 5.32E-05 |
| ST7-OT4 | 5.32E-05 |
| WT1-AS | 5.39E-05 |
| FLG-AS1 | 5.40E-05 |
| C22orf34 | 5.41E-05 |
| SHANK3 | 5.41E-05 |
| BBOX1-AS1 | 5.41E-05 |
| DSG2-AS1 | 5.50E-05 |
| MAP3K14-AS1 | 5.54E-05 |
| LOC388780 | 5.56E-05 |
| MAPKAPK5-AS1 | 5.59E-05 |
| PITRM1-AS1 | 5.61E-05 |
| CCDC13-AS1 | 5.62E-05 |
| LINC01422 | 5.66E-05 |
| OXCT1-AS1 | 5.71E-05 |
| PCOLCE-AS1 | 5.77E-05 |
| ZMIZ1-AS1 | 5.78E-05 |
| TRIM52-AS1 | 5.83E-05 |
| KCNMA1-AS1 | 5.86E-05 |
| STK4-AS1 | 5.95E-05 |
| LINC01126 | 6.02E-05 |
| TTC39C-AS1 | 6.08E-05 |
| CACNA1C-AS2 | 6.42E-05 |
| CACNA1C-AS1 | 6.42E-05 |
| ZNF197-AS1 | 6.46E-05 |
| LINC00174 | 6.57E-05 |
| LOC100507283 | 6.60E-05 |
| LINC01144 | 6.62E-05 |
| STT3A-AS1 | 6.62E-05 |
| CFLAR-AS1 | 6.76E-05 |
| LOC256880 | 6.92E-05 |
| TTC3-AS1 | 6.97E-05 |
| DSCR9 | 6.97E-05 |
| LOC102724009 | 7.00E-05 |
| RAB11B-AS1 | 7.01E-05 |
| LINC01372 | 7.03E-05 |
| LOC102724589 | 7.11E-05 |
| PAX8-AS1 | 7.14E-05 |
| LOC100128494 | 7.19E-05 |
| IDI2-AS1 | 7.21E-05 |
| MIR3142HG | 7.23E-05 |
| LINC00857 | 7.32E-05 |
| LOC642361 | 7.32E-05 |
| NUTM2B-AS1 | 7.32E-05 |
| MCM8-AS1 | 7.47E-05 |
| LINC01182 | 7.48E-05 |
| MORC2-AS1 | 7.92E-05 |
| AFAP1-AS1 | 7.95E-05 |
| PAXBP1-AS1 | 8.04E-05 |
| DISC1FP1 | 8.08E-05 |
| UPK1A-AS1 | 8.09E-05 |
| LINC01529 | 8.09E-05 |
| LOC102724532 | 8.10E-05 |
| LINC01224 | 8.18E-05 |
| TTC39A-AS1 | 8.24E-05 |
| LINC01370 | 8.37E-05 |
| LOC101927377 | 8.54E-05 |
| LINC00261 | 8.57E-05 |
| LOC100507564 | 8.71E-05 |
| HOXB-AS1 | 8.91E-05 |
| HOXB-AS3 | 8.91E-05 |
| DTX2P1-UPK3BP1-PMS2P11 | 8.96E-05 |
| LINC00664 | 9.03E-05 |
| LOH12CR2 | 9.12E-05 |
| PAXIP1-AS2 | 9.12E-05 |
| PAXIP1-AS1 | 9.12E-05 |
| AGAP2-AS1 | 9.18E-05 |
| LOC103344931 | 9.18E-05 |
| ADAMTS9-AS1 | 9.19E-05 |
| A2M-AS1 | 9.33E-05 |
| PIK3CD-AS1 | 9.35E-05 |
| PIK3CD-AS2 | 9.35E-05 |
| LOC375196 | 9.37E-05 |
| LINC01320 | 9.50E-05 |
| LOC101929089 | 9.52E-05 |
| LINC01118 | 9.61E-05 |
| LINC01119 | 9.61E-05 |
| LINC01315 | 9.84E-05 |
| LOC100506679 | 0.000101402 |
| DGCR11 | 0.000102613 |
| DGCR9 | 0.000102613 |
| LINC00896 | 0.000102613 |
| LOC284865 | 0.000102613 |
| THAP7-AS1 | 0.000102613 |
| TMEM191A | 0.000102613 |
| TMEM191C | 0.000102613 |
| APTR | 0.000104444 |
| PTPRG-AS1 | 0.000104467 |
| CACNA1G-AS1 | 0.000106273 |
| LOC101927043 | 0.000107491 |
| LOC100128239 | 0.000109543 |
| SATB2-AS1 | 0.000109776 |
| LOC101926943 | 0.000114668 |
| LINC00964 | 0.000116554 |
| LINC00662 | 0.000119251 |
| LOC101927151 | 0.000119251 |
| LINC00494 | 0.000120697 |
| LINC00310 | 0.000122797 |
| SNHG22 | 0.000123799 |
| UCA1 | 0.000125021 |
| UGDH-AS1 | 0.000126575 |
| TP53TG1 | 0.000127215 |
| HPN-AS1 | 0.000128269 |
| LOC100996351 | 0.00012945 |
| EPHA1-AS1 | 0.0001298 |
| LINC-PINT | 0.00013056 |
| UBE2Q1-AS1 | 0.000130616 |
| PRKAR2A-AS1 | 0.000131071 |
| ALMS1-IT1 | 0.000133813 |
| SCARNA17 | 0.000133923 |
| MIR22HG | 0.000134167 |
| GUSBP11 | 0.000134659 |
| CYP1B1-AS1 | 0.000136059 |
| LOC103611081 | 0.000137822 |
| LOC104968399 | 0.000139642 |
| LINC00504 | 0.00013996 |
| LOC101930071 | 0.000142851 |
| C20orf203 | 0.000144227 |
| LOC101559451 | 0.00014631 |
| TMEM147-AS1 | 0.000148807 |
| PLCG1-AS1 | 0.000156411 |
| LOC105371485 | 0.000157574 |
| GDNF-AS1 | 0.000157601 |
| LOC100129931 | 0.000158476 |
| LINC01589 | 0.000160541 |
| LOC100131496 | 0.000166402 |
| LOC400655 | 0.000167437 |
| LOC200772 | 0.000171602 |
| LINC01426 | 0.000171943 |
| LOC101929698 | 0.000177701 |
| ASH1L-AS1 | 0.000178015 |
| ZNF341-AS1 | 0.000178266 |
| MAGI2-AS3 | 0.000178995 |
| RBM5-AS1 | 0.000179319 |
| SEMA3B-AS1 | 0.000179385 |
| LINC00649 | 0.000185795 |
| LINC00987 | 0.000191731 |
| LOC101927571 | 0.000193539 |
| POT1-AS1 | 0.000195214 |
| LINC01393 | 0.000195676 |
| LINC01265 | 0.000201003 |
| ADORA2A-AS1 | 0.0002041 |
| SNHG17 | 0.000207783 |
| LOC105373805 | 0.00021778 |
| UTAT33 | 0.000224558 |
| LOC105371824 | 0.000234079 |
| TMEM92-AS1 | 0.000240453 |
| LOC101929626 | 0.00024058 |
| LINC00189 | 0.000240717 |
| STEAP2-AS1 | 0.000251335 |
| LOC101927543 | 0.000256032 |
| LDLRAD4-AS1 | 0.000256169 |
| C3orf67-AS1 | 0.000256426 |
| LRRC2-AS1 | 0.000257028 |
| STPG3-AS1 | 0.000257744 |
| LOC100133286 | 0.000261222 |
| CBR3-AS1 | 0.000261222 |
| EGFR-AS1 | 0.000261931 |
| LOC103091866 | 0.000262797 |
| LOC101927550 | 0.000269883 |
| LOC101927394 | 0.000270833 |
| LOC100507053 | 0.000273475 |
| PITPNA-AS1 | 0.000274666 |
| CYP4F35P | 0.00028047 |
| APOA1-AS | 0.000288057 |
| RUSC1-AS1 | 0.000289376 |
| GATA6-AS1 | 0.000291153 |
| LOC100287792 | 0.000295874 |
| LOC101929574 | 0.000296158 |
| LOC102724596 | 0.000314487 |
| LOC101928424 | 0.000318686 |
| LOC102723838 | 0.000318686 |
| FOXD2-AS1 | 0.000326576 |
| C20orf197 | 0.000338947 |
| LOC101927420 | 0.000340167 |
| LOC171391 | 0.000344674 |
| LINC01473 | 0.000344765 |
| SP2-AS1 | 0.000347279 |
| MIRLET7BHG | 0.000352676 |
| ASAP1-IT2 | 0.000354942 |
| ABALON | 0.000364612 |
| LOC100506302 | 0.000376916 |
| SUCLG2-AS1 | 0.000381504 |
| LOC101928834 | 0.00038304 |
| PSMD6-AS2 | 0.000383392 |
| LIFR-AS1 | 0.000388158 |
| LINC00672 | 0.000389453 |
| CA3-AS1 | 0.000390064 |
| TET3 | 0.000390666 |
| BOLA3-AS1 | 0.000390666 |
| ITIH4-AS1 | 0.000394391 |
| LINC00909 | 0.00039483 |
| KANSL1-AS1 | 0.000399909 |
| MAPT-IT1 | 0.000399909 |
| LOC101929536 | 0.000405494 |
| LOC100507670 | 0.000408145 |
| HNF4A-AS1 | 0.000408976 |
| GCC2-AS1 | 0.00041467 |
| LOC105371907 | 0.000418486 |
| LINC00854 | 0.000423986 |
| LINC00910 | 0.000423986 |
| THUMPD3-AS1 | 0.000426734 |
| LOC101927596 | 0.000432094 |
| KC6 | 0.000434048 |
| PVT1 | 0.000435591 |
| LINC01503 | 0.000436347 |
| LINC01587 | 0.000440283 |
| CYB561D2 | 0.000442569 |
| NIFK-AS1 | 0.000443534 |
| LINC01237 | 0.000460125 |
| LOC285095 | 0.000460125 |
| LINC00114 | 0.000483322 |
| LOC100506388 | 0.000485103 |
| LOC101927727 | 0.000485103 |
| NKILA | 0.00049274 |
| FAM3D-AS1 | 0.000496771 |
| RASSF1-AS1 | 0.000498806 |
| STEAP3-AS1 | 0.000510463 |
| LOC151174 | 0.000513231 |
| LOC100128317 | 0.000519442 |
| LOC100130691 | 0.000526064 |
| ARF4-AS1 | 0.000528339 |
| LOC101927588 | 0.000530651 |
| RNF139-AS1 | 0.000530651 |
| HM13-AS1 | 0.000536569 |
| LOC101929532 | 0.000545322 |
| GHRLOS | 0.000547974 |
| LINC00852 | 0.000547974 |
| LINC01270 | 0.000561443 |
| LINC01271 | 0.000561443 |
| LINC00861 | 0.000570306 |
| TNRC6C-AS1 | 0.000577904 |
| FAM225A | 0.000580702 |
| FAM225B | 0.000580702 |
| ZNF883 | 0.000580702 |
| NCRNA00250 | 0.000583324 |
| LINC01123 | 0.000583972 |
| LOC100507334 | 0.000583972 |
| LOC440895 | 0.000583972 |
| DGUOK-AS1 | 0.000584294 |
| LOC101929066 | 0.00058434 |
| GLIDR | 0.000588511 |
| LINC01410 | 0.000588511 |
| LOC728673 | 0.000588511 |
| PGM5-AS1 | 0.000588511 |
| PGM5P2 | 0.000588511 |
| SEMA3F-AS1 | 0.000590409 |
| MIR600HG | 0.000600507 |
| LOC101928674 | 0.000602018 |
| MIR4435-2HG | 0.000604884 |
| GNAS-AS1 | 0.000625241 |
| LINC01151 | 0.000630663 |
| LOC101927989 | 0.000660391 |
| LINC00908 | 0.000660391 |
| LINC00683 | 0.000660391 |
| LINC00624 | 0.000664587 |
| LINC00869 | 0.000664587 |
| LINC01138 | 0.000664587 |
| LOC100505824 | 0.000664587 |
| SNHG7 | 0.000683207 |
| UMAD1 | 0.000683805 |
| PARD6G-AS1 | 0.00068387 |
| RBFADN | 0.00068387 |
| PRRT3-AS1 | 0.000693342 |
| SLC22A18AS | 0.000713129 |
| RALY-AS1 | 0.000727207 |
| LOC101928881 | 0.000729656 |
| TOLLIP-AS1 | 0.000742137 |
| CASC11 | 0.000752867 |
| C1QTNF1-AS1 | 0.000759337 |
| LBX2-AS1 | 0.000765169 |
| DBH-AS1 | 0.000771143 |
| LAMA5-AS1 | 0.000773449 |
| LINC01152 | 0.000779116 |
| LOC102723517 | 0.000779116 |
| PCAT19 | 0.000779151 |
| LINC01480 | 0.000779151 |
| ISPD-AS1 | 0.000785006 |
| LINC00511 | 0.000797987 |
| SEPT4-AS1 | 0.000798612 |
| THCAT158 | 0.000800674 |
| OGFR-AS1 | 0.000807804 |
| ZNF529-AS1 | 0.000812829 |
| LOC100505715 | 0.000816999 |
| LOC101928063 | 0.000816999 |
| LOC100379224 | 0.000816999 |
| LOC93622 | 0.000839025 |
| HSPC324 | 0.000842991 |
| ELFN1-AS1 | 0.000844558 |
| MIR181A2HG | 0.00084762 |
| LINC00907 | 0.000849807 |
| SLC6A1-AS1 | 0.00087804 |
| SPATA3-AS1 | 0.000882787 |
| LOC101929054 | 0.000889187 |
| TOB1-AS1 | 0.000891866 |
| MIR210HG | 0.000896315 |
| ARRDC1-AS1 | 0.000899816 |
| LINC01446 | 0.000915555 |
| LOC101927572 | 0.000923326 |
| UCKL1-AS1 | 0.000980637 |
| LMCD1-AS1 | 0.00098719 |
| SOX9-AS1 | 0.000997694 |
| MIR646HG | 0.00100032 |
| PTGES2-AS1 | 0.001002079 |
| PCBP1-AS1 | 0.001031332 |
| LINC00667 | 0.001035942 |
| FALEC | 0.001047232 |
| LINC01451 | 0.001049093 |
| LOC101928103 | 0.001049386 |
| PPP1R26-AS1 | 0.001051265 |
| LOC101928525 | 0.001051265 |
| ADNP-AS1 | 0.001060587 |
| LOC101928797 | 0.001060803 |
| ZFAS1 | 0.001061377 |
| SLCO4A1-AS1 | 0.001085306 |
| CASC8 | 0.001095111 |
| TSPOAP1-AS1 | 0.001095916 |
| LINC00665 | 0.001106238 |
| RAD21-AS1 | 0.001137812 |
| DLGAP4-AS1 | 0.001157034 |
| PSMG3-AS1 | 0.00116745 |
| LINC01606 | 0.001177351 |
| LOC101927503 | 0.001187838 |
| GAPLINC | 0.001193516 |
| LINC00313 | 0.00126756 |
| H19 | 0.001301164 |
| MRPL23-AS1 | 0.001301164 |
| IGF2-AS | 0.001301164 |
| LOC728752 | 0.00130921 |
| LINC01534 | 0.00130921 |
| LOC728485 | 0.00130921 |
| ZNF790-AS1 | 0.00130921 |
| TTN-AS1 | 0.00135526 |
| HAR1B | 0.001362364 |
| LOC101927497 | 0.00138213 |
| SNHG20 | 0.001413198 |
| LINC01291 | 0.001425008 |
| DKFZP434A062 | 0.001425643 |
| SNHG6 | 0.001458817 |
| KRTAP5-AS1 | 0.001486257 |
| RARA-AS1 | 0.00150421 |
| NORAD | 0.001545692 |
| MIR155HG | 0.001551026 |
| LINC00515 | 0.001551026 |
| RRS1-AS1 | 0.001559808 |
| LOC101928403 | 0.001568365 |
| NDUFV2-AS1 | 0.001592572 |
| LOC729867 | 0.001592974 |
| KCNQ1OT1 | 0.001602003 |
| LOC101927661 | 0.001617345 |
| LOC101927630 | 0.001633959 |
| HRAT92 | 0.001636394 |
| LOC100506497 | 0.001653138 |
| LINC00323 | 0.001681995 |
| LOC105371849 | 0.00168793 |
| WFDC21P | 0.00168793 |
| YTHDF3-AS1 | 0.001695078 |
| LOC339803 | 0.001696137 |
| LOC100505622 | 0.001707245 |
| LOC101927196 | 0.001708652 |
| LOC102723322 | 0.001713368 |
| LOC101929231 | 0.001716428 |
| LINC01474 | 0.001719423 |
| RGS5 | 0.001730866 |
| LOC105372833 | 0.001809554 |
| LINC00483 | 0.001842297 |
| LOC105371730 | 0.001845893 |
| CYP51A1-AS1 | 0.001847157 |
| LINC01133 | 0.001895394 |
| LOC100506082 | 0.001905719 |
| SMC2-AS1 | 0.001910199 |
| LINC01506 | 0.001942403 |
| GSN-AS1 | 0.001979906 |
| LINC01273 | 0.001994237 |
| LOC101927811 | 0.002006435 |
| RAMP2-AS1 | 0.002044963 |
| SH3BP5-AS1 | 0.002075996 |
| C5orf17 | 0.002115174 |
| CSE1L-AS1 | 0.002146297 |
| LOC101927884 | 0.002148009 |
| LOC105375166 | 0.002172295 |
| ZNF252P-AS1 | 0.002277161 |
| LINC01535 | 0.002299413 |
| LINC01504 | 0.002312936 |
| LOC100506100 | 0.002326139 |
| TEX41 | 0.002335248 |
| MAMDC2-AS1 | 0.002354445 |
| ASB16-AS1 | 0.002373019 |
| LOC100506178 | 0.002377072 |
| LOC286178 | 0.002419374 |
| MGC16275 | 0.002426399 |
| BHLHE40-AS1 | 0.002434294 |
| TYMSOS | 0.002453701 |
| FAM83A-AS1 | 0.002471775 |
| LOC101928372 | 0.002497039 |
| LOC101929331 | 0.002500335 |
| TMEM246-AS1 | 0.00252716 |
| LOC100130587 | 0.002552344 |
| LOC101927027 | 0.002576683 |
| HAGLR | 0.002580568 |
| LOC101927055 | 0.002588259 |
| LINC01447 | 0.002621009 |
| BAZ2B | 0.002638964 |
| CEBPB-AS1 | 0.002684854 |
| KCNJ2-AS1 | 0.002700289 |
| ZNF385C | 0.002710155 |
| LOC286059 | 0.002712884 |
| LINC00471 | 0.002725372 |
| PRR29-AS1 | 0.002737021 |
| SNHG25 | 0.002737021 |
| FGD5-AS1 | 0.002769129 |
| LOC101929709 | 0.00277577 |
| LOC101927018 | 0.002800365 |
| ZNF571-AS1 | 0.002815335 |
| LOC105274304 | 0.002828221 |
| FAM83C-AS1 | 0.002835889 |
| LINC00671 | 0.002858698 |
| EGOT | 0.002915688 |
| LINC01549 | 0.002955959 |
| LINC01124 | 0.003001903 |
| ERICH2 | 0.003001903 |
| LINC00525 | 0.003017024 |
| AGAP1-IT1 | 0.003100789 |
| CDKN2B-AS1 | 0.003110658 |
| LOC389641 | 0.003226584 |
| LOC100507156 | 0.003226584 |
| MIR924HG | 0.003392717 |
| LOC730338 | 0.00348663 |
| LOC101927402 | 0.003515204 |
| FAM99A | 0.003524861 |
| FAM99B | 0.003524861 |
| CD81-AS1 | 0.003524861 |
| LOC101929282 | 0.003586668 |
| LOC101930085 | 0.003681889 |
| LOC100506725 | 0.003681889 |
| DARS-AS1 | 0.003752845 |
| AATBC | 0.003789429 |
| LOC100287042 | 0.003807294 |
| ZNF295-AS1 | 0.003911671 |
| SLFNL1-AS1 | 0.003923563 |
| LINC01117 | 0.003952874 |
| LINC00479 | 0.003953958 |
| LOC100294362 | 0.004007053 |
| ITGB2-AS1 | 0.004046003 |
| LINC01547 | 0.004046003 |
| TSPEAR-AS1 | 0.004113575 |
| LINC01482 | 0.004115372 |
| VLDLR-AS1 | 0.004182834 |
| LINC01176 | 0.00430037 |
| PCAT1 | 0.004308982 |
| PCAT2 | 0.004308982 |
| CCDC183-AS1 | 0.004315294 |
| LENG8-AS1 | 0.004331389 |
| LOC100506098 | 0.004425404 |
| ZFHX4-AS1 | 0.004449383 |
| MIR2052HG | 0.004542769 |
| SNHG16 | 0.004591811 |
| LOC105375304 | 0.004619223 |
| LOC441204 | 0.004619223 |
| HOTAIRM1 | 0.004619223 |
| HOXA-AS3 | 0.004619223 |
| HOXA11-AS | 0.004619223 |
| RFX3-AS1 | 0.00462628 |
| HDAC11-AS1 | 0.004682958 |
| EXTL3-AS1 | 0.00473769 |
| LINC01419 | 0.004747628 |
| LOC101928673 | 0.004784029 |
| TRG-AS1 | 0.004800558 |
| LINC00506 | 0.004952361 |
| LOC101927855 | 0.004994242 |
| TBX2-AS1 | 0.004994242 |
| INHBA-AS1 | 0.005007758 |
| GAS5-AS1 | 0.005053869 |
| GAS5 | 0.005053869 |
| LOC102724601 | 0.005053869 |
| LOC102724058 | 0.005085253 |
| LOC101929680 | 0.005085253 |
| LOC105375800 | 0.005106346 |
| STAU2-AS1 | 0.005296486 |
| COL18A1-AS2 | 0.00536867 |
| LINC00334 | 0.00536867 |
| DNM3OS | 0.005387524 |
| LOC101928565 | 0.005401041 |
| LOC101929552 | 0.005433922 |
| ZEB2-AS1 | 0.005452772 |
| RAPGEF4-AS1 | 0.005587401 |
| PPP4R1-AS1 | 0.005636466 |
| RNF157-AS1 | 0.005686333 |
| LOC401463 | 0.005733564 |
| POU6F2-AS1 | 0.00587322 |
| LOC101929268 | 0.005874695 |
| LINC01607 | 0.00599524 |
| ALDH1L1-AS1 | 0.006013146 |
| LINC00488 | 0.006241911 |
| LOC100507071 | 0.00624634 |
| LOC100130548 | 0.00635249 |
| ADAMTSL4-AS1 | 0.006454643 |
| RBPMS-AS1 | 0.006699043 |
| UBR5-AS1 | 0.006760419 |
| NPSR1-AS1 | 0.006772206 |
| LOC100129917 | 0.006844149 |
| LOC653712 | 0.006920426 |
| FAM83H-AS1 | 0.006968264 |
| HID1-AS1 | 0.007041575 |
| LOC101929128 | 0.007046547 |
| LOC157273 | 0.007046547 |
| IDH1-AS1 | 0.007124904 |
| LOC101927040 | 0.007132272 |
| LOC101927502 | 0.007182252 |
| MINCR | 0.007196071 |
| RHPN1-AS1 | 0.007196071 |
| ALDH1L1-AS2 | 0.00726353 |
| RDH10-AS1 | 0.007266805 |
| LOC101927056 | 0.007388711 |
| LINC01239 | 0.007652254 |
| SPACA6 | 0.00771438 |
| MAFA-AS1 | 0.007741456 |
| ZNF582-AS1 | 0.007808695 |
| LOC105373878 | 0.007816261 |
| ABCA9-AS1 | 0.007982046 |
| LOC101928161 | 0.008179812 |
| LOC100132215 | 0.008309677 |
| CFAP99 | 0.008351873 |
| LINC00607 | 0.008437495 |
| LINC01132 | 0.008655985 |
| CTBP1-AS | 0.008944623 |
| ZNF667-AS1 | 0.009312403 |
| ZIM2-AS1 | 0.009312403 |
| LOC105376114 | 0.00947505 |
| A1BG-AS1 | 0.009481522 |
| LOC105372483 | 0.009481522 |
| MZF1-AS1 | 0.009481522 |
| LINC00535 | 0.009793066 |
| GAS1RR | 0.009854805 |
| LOC101927851 | 0.010268161 |
| MSC-AS1 | 0.010423045 |
| SNHG15 | 0.010550952 |
| LACTB2-AS1 | 0.010961953 |
| STX17-AS1 | 0.011147126 |
| STX18-AS1 | 0.011262541 |
| ARHGAP31-AS1 | 0.011635119 |
| LOC102723582 | 0.011785185 |
| LINCR-0001 | 0.011826445 |
| C8orf31 | 0.01184301 |
| LOC100128398 | 0.011860213 |
| LINC01215 | 0.011882005 |
| ZNF350-AS1 | 0.012197663 |
| ZNF649-AS1 | 0.012197663 |
| IPO9-AS1 | 0.012337618 |
| IFT74-AS1 | 0.012404461 |
| MIR4458HG | 0.012794227 |
| LINC00680 | 0.01352469 |
| EBLN3P | 0.013597868 |
| LOC102723566 | 0.013682369 |
| LOC654841 | 0.013713664 |
| FOCAD-AS1 | 0.013967583 |
| LOC101926892 | 0.014376709 |
| ST3GAL6-AS1 | 0.015011502 |
| LINC00882 | 0.015135884 |
| DUBR | 0.015135884 |
| C1orf140 | 0.015228573 |
| LOC93429 | 0.015307717 |
| ZBTB11-AS1 | 0.015396819 |
| SNHG18 | 0.015417141 |
| LOC100288181 | 0.015498094 |
| CERS6-AS1 | 0.015742912 |
| LOC101928438 | 0.016455047 |
| CARD8-AS1 | 0.016481655 |
| ZNF888 | 0.016655289 |
| DACT3-AS1 | 0.017321734 |
| LOC339874 | 0.01800455 |
| NPHP3-AS1 | 0.018418505 |
| MCM3AP-AS1 | 0.018516261 |
| LINC01139 | 0.018666306 |
| OTUD6B-AS1 | 0.019094579 |
| LOC101929224 | 0.019224485 |
| ZBTB20-AS1 | 0.019637836 |
| MIR4290HG | 0.019850768 |
| LOC100505635 | 0.020133405 |
| CATIP-AS1 | 0.020205089 |
| LINC00862 | 0.020279984 |
| TMCC1-AS1 | 0.022441517 |
| NOP14-AS1 | 0.022947596 |
| LOC101928307 | 0.023569915 |
| LGALS8-AS1 | 0.023741834 |
| LOC101927604 | 0.024930694 |
| RNF217-AS1 | 0.025214386 |
| LOC100129175 | 0.025609926 |
| LOC101928517 | 0.026005012 |
| PCAT7 | 0.027612426 |
| FLJ46284 | 0.028054145 |
| LINC00628 | 0.028152107 |
| LINC00184 | 0.028540423 |
| LOC101927787 | 0.028540423 |
| POLR2J4 | 0.028713863 |
| LOC100507557 | 0.028742244 |
| BAALC-AS2 | 0.028964084 |
| BAALC-AS1 | 0.028964084 |
| RAET1E-AS1 | 0.029691762 |
| MYLK-AS1 | 0.030988904 |
| CHRM3-AS2 | 0.034321767 |
| HLX-AS1 | 0.034635476 |
| LINC01352 | 0.034635476 |
| LOC102723701 | 0.035406063 |
| LOC100287015 | 0.035858476 |
| LOC101927765 | 0.035995488 |
| LINC00476 | 0.036435379 |
| LINC00092 | 0.036435379 |
| LINC01251 | 0.036631102 |
| MIRLET7DHG | 0.037322034 |
| LOC100132077 | 0.037322034 |
| LINC01354 | 0.037348066 |
| UNQ6494 | 0.037806366 |
| PSORS1C3 | 0.038670685 |
| C1orf220 | 0.039329911 |
| LINC01625 | 0.040710965 |
| NAPA-AS1 | 0.041094399 |
| RASAL2-AS1 | 0.041260244 |
| LINC01018 | 0.041733207 |
| PTOV1-AS1 | 0.043778661 |
| PLA2G4C-AS1 | 0.04397652 |
| CPS1-IT1 | 0.044914088 |
| B4GALT1-AS1 | 0.045356705 |
| LOC400710 | 0.046120352 |
| FAM66B | 0.048528452 |
| TSTD3 | 0.050557903 |
| C8orf37-AS1 | 0.050667912 |
| LOC100130476 | 0.051776438 |
| LOC105372440 | 0.052298335 |
| LINC00473 | 0.052771764 |
| NCOA7-AS1 | 0.05371602 |
| DSE | 0.054891589 |
| LURAP1L-AS1 | 0.063461597 |
| TGFB2-AS1 | 0.064011853 |
| LINC00271 | 0.064603698 |
| LOC101927136 | 0.064852506 |
| LOC101927189 | 0.067275255 |
| LOC100507291 | 0.067465362 |
| HMGN3-AS1 | 0.068267926 |
| KLHL6-AS1 | 0.069376838 |
| LOC153910 | 0.070621739 |
| LINC01016 | 0.071220809 |
| ZNF528-AS1 | 0.071964269 |
| FAM87A | 0.073570906 |
| HCG9 | 0.073597316 |
| ZNRD1ASP | 0.073597316 |
| LOC101927752 | 0.074259817 |
| MLIP-IT1 | 0.077563944 |
| HCG14 | 0.079289462 |
| KBTBD11-OT1 | 0.081196539 |
| LINC01353 | 0.083068369 |
| LINC01136 | 0.083068369 |
| TRIM31-AS1 | 0.084524371 |
| HCG25 | 0.086302134 |
| LINC01615 | 0.087791277 |
| LOC101929523 | 0.087791277 |
| LINC00242 | 0.089527741 |
| LINC00574 | 0.089527741 |
| PCAT6 | 0.090415874 |
| LOC148709 | 0.090415874 |
| SMG7-AS1 | 0.091823401 |
| LINC00240 | 0.091874847 |
| LINC01564 | 0.094079754 |
| HCG22 | 0.094638156 |
| C6orf99 | 0.095408221 |
| LINC00501 | 0.095498298 |
| LINC01063 | 0.10022195 |
| LOC339529 | 0.101061193 |
| C2-AS1 | 0.106665421 |
| DDX39B-AS1 | 0.106665421 |
| HCG23 | 0.106665421 |
| HCP5 | 0.106665421 |
| HLA-DQB1-AS1 | 0.106665421 |
| PSMB8-AS1 | 0.106665421 |
| LOC100506688 | 0.106831471 |
| LOC101927143 | 0.107967541 |
| LOC101927164 | 0.107967541 |
| LINC01013 | 0.11093679 |
| LOC101928295 | 0.111968073 |
| LINC01558 | 0.114601293 |
| FOXP4-AS1 | 0.114968374 |
| TARID | 0.115542146 |
| HLA-F-AS1 | 0.119535385 |
| TRAM2-AS1 | 0.12579558 |
| TRAF3IP2-AS1 | 0.125944149 |
| TAF1A-AS1 | 0.126446915 |
| LOC100507389 | 0.133626401 |
| PAQR9-AS1 | 0.133626401 |
| TM4SF19-AS1 | 0.137842186 |
| EXOC3-AS1 | 0.141401414 |
| PP7080 | 0.141401414 |
| CTD-3080P12.3 | 0.141595213 |
| LINC00881 | 0.142291569 |
| OSTN-AS1 | 0.143656366 |
| LINC01268 | 0.144932237 |
| LINC00885 | 0.148251085 |
| HCG20 | 0.148654109 |
| LINC00243 | 0.148654109 |
| LINC01344 | 0.151346799 |
| DLG1-AS1 | 0.154012207 |
| KIF25-AS1 | 0.156141234 |
| LOC101929420 | 0.156141234 |
| NCBP2-AS2 | 0.159404349 |
| SDHAP2 | 0.160201092 |
| LINC01012 | 0.163528057 |
| XXYLT1-AS2 | 0.166028727 |
| LOC105374952 | 0.17886439 |
| LINC00886 | 0.181568086 |
| LOC100289361 | 0.182615512 |
| LINC00222 | 0.184848671 |
| LOC102723727 | 0.185038132 |
| HCG11 | 0.188588622 |
| LOC100506885 | 0.198764374 |
| LOC102724804 | 0.202159534 |
| LOC101929541 | 0.203452 |
| LOC102724919 | 0.204588853 |
| LOC101929705 | 0.205498091 |
| ZSCAN16-AS1 | 0.208720653 |
| CASC15 | 0.214629283 |
| HCG17 | 0.219109947 |
| SNHG5 | 0.227144518 |
| LOC101929243 | 0.233861153 |
| LINC00880 | 0.236993744 |
| PACRG-AS1 | 0.24078883 |
| TM4SF1-AS1 | 0.24849448 |
| CAHM | 0.254008249 |
| LINC00887 | 0.263439394 |
| HULC | 0.268167568 |
| CLRN1-AS1 | 0.270690062 |
| LINC01108 | 0.271887815 |
| LOC100507506 | 0.295218124 |
| LYRM4-AS1 | 0.295218124 |
| IGF2BP2-AS1 | 0.312251755 |
| JARID2-AS1 | 0.323560386 |
| TPRG1-AS1 | 0.326402956 |
| LOC101927972 | 0.331554753 |
| LPP-AS2 | 0.3316553 |
| LOC100130357 | 0.333445926 |
| SERTAD4-AS1 | 0.351116035 |
| ARHGEF26-AS1 | 0.357938141 |
| ERICH6-AS1 | 0.378558096 |
| WWTR1-AS1 | 0.391541027 |
| LOC101930114 | 0.450841899 |
| LY86-AS1 | 0.453592709 |
| MBNL1-AS1 | 0.490616915 |
