# Supplementary material for: Four-copy number alteration (CNA)-related lncRNA prognostic signature for liver cancer
Source: Sci Rep. 2022 Aug 22;12:14261. doi: 10.1038/s41598-022-17927-0 (PMC9395537; doi:10.1038/s41598-022-17927-0)
Supplement: Supplementary file 3 — Supplementary Table 2. [file 41598_2022_17927_MOESM3_ESM.docx]

Supplementary Table 2: The differences in the expression of these 52 lncRNAs with copy number gain, copy number loss, and normal copy number.

| lncRNA | median | lower.95 | up.95 | p.value |
| --- | --- | --- | --- | --- |
| LOC101929066 | 0.144683002 | 0.178601857 | 0.216131514 | 3.06E-16 |
| RUSC1-AS1 | 1.175509276 | 1.183868813 | 1.315598547 | 8.88E-12 |
| RGS5 | 0.297960558 | 0.321997703 | 0.366685275 | 1.43E-10 |
| GAS5 | 4.593018691 | 4.537430854 | 4.776338351 | 1.72E-10 |
| LOC148709 | 0.377985189 | 0.51012594 | 0.622551624 | 8.67E-09 |
| LOC101928673 | 0.457377148 | 0.503810326 | 0.570160895 | 3.82E-08 |
| LOC389641 | 0.510778989 | 0.543142706 | 0.618165214 | 5.07E-08 |
| TAF1A-AS1 | 1.129550593 | 1.160917521 | 1.274227818 | 1.75E-07 |
| LINC01136 | 0.07034954 | 0.094196176 | 0.127760492 | 1.89E-06 |
| FALEC | 0.144327258 | 0.172732366 | 0.2116578 | 3.15E-06 |
| PCAT6 | 1.320557254 | 1.311341462 | 1.471400832 | 4.89E-06 |
| LOC100507670 | 1.248986327 | 1.289094036 | 1.422501294 | 8.18E-06 |
| FAM66B | 0.015972771 | 0.03003229 | 0.044878488 | 1.60E-05 |
| ASH1L-AS1 | 0.691689192 | 0.721602998 | 0.802979449 | 2.87E-05 |
| LOC101929224 | 0.137109384 | 0.249309941 | 0.36815696 | 3.71E-05 |
| GAS5-AS1 | 1.009604224 | 1.036775436 | 1.119200372 | 5.49E-05 |
| EXTL3-AS1 | 0.085296367 | 0.10421548 | 0.125705969 | 8.12E-05 |
| LOC100507071 | 0.2035227 | 0.220540202 | 0.280549735 | 0.000202026 |
| LOC101927851 | 1.078787149 | 1.162885545 | 1.307387858 | 0.000235606 |
| SMG7-AS1 | 0.112904104 | 0.12313809 | 0.141457727 | 0.000280115 |
| C1orf220 | 0.258530516 | 0.286673155 | 0.34008824 | 0.000398783 |
| LOC101928565 | 0.217645593 | 0.3151784 | 0.411375648 | 0.000537577 |
| LOC101929128 | 0.492142627 | 0.615777164 | 0.763389573 | 0.00057245 |
| LOC102724601 | 0.148838903 | 0.183560912 | 0.224252134 | 0.000577759 |
| LINC00862 | 0.186731381 | 0.276316442 | 0.354144031 | 0.000646466 |
| LOC102724919 | 0.185398526 | 0.203604125 | 0.241909061 | 0.000796527 |
| IPO9-AS1 | 0.114004839 | 0.126849715 | 0.151620855 | 0.000967297 |
| LINC01344 | 0.101121764 | 0.160748437 | 0.206923179 | 0.001553029 |
| ADAMTSL4-AS1 | 0.126293008 | 0.138234975 | 0.159739646 | 0.001949833 |
| LOC101928372 | 0.012536512 | 0.026395155 | 0.040413412 | 0.002401766 |
| UBE2Q1-AS1 | 0.190685227 | 0.222501134 | 0.274306786 | 0.003530491 |
| LINC01353 | 0.100547309 | 0.162168485 | 0.212776967 | 0.003749696 |
| LOC101929541 | 1.78453102 | 1.877622839 | 2.186936686 | 0.004976308 |
| LOC729867 | 0.113895109 | 0.123407662 | 0.143157925 | 0.011244911 |
| LOC286059 | 0.010056269 | 0.011132393 | 0.014567576 | 0.021949267 |
| LINC01133 | 0.044347604 | 0.086695397 | 0.152243793 | 0.026066494 |
| LINC00628 | 0.085723527 | 0.121901159 | 0.156113059 | 0.041953155 |
| DNM3OS | 0.128778356 | 0.175779997 | 0.222830225 | 0.05281277 |
| LOC157273 | 0.320819451 | 0.586720095 | 0.742515726 | 0.07336036 |
| RASAL2-AS1 | 0.250257343 | 0.275083822 | 0.317799545 | 0.077901383 |
| LINCR-0001 | 0.167746013 | 0.311487573 | 0.422526933 | 0.081034698 |
| LOC101930114 | 1.584222084 | 1.595771915 | 1.822790085 | 0.119491035 |
| LINC01352 | 0.24004369 | 0.270241168 | 0.332238347 | 0.164345935 |
| HLX-AS1 | 0.060185332 | 0.107259689 | 0.144952347 | 0.263041637 |
| LINC01354 | 0.080958156 | 0.129391002 | 0.172756459 | 0.296204692 |
| LOC100507156 | 0.029343951 | 0.036078129 | 0.04493668 | 0.296760487 |
| LINC00184 | 0.012397486 | 0.019016653 | 0.026827945 | 0.315496546 |
| TGFB2-AS1 | 0.117825037 | 0.238683561 | 0.342348973 | 0.492428685 |
| C1orf140 | 0.008525653 | 0.072730063 | 0.119750451 | 0.557499048 |
| LINC01132 | 0.139409878 | 0.17693575 | 0.215140955 | 0.604382988 |
| LOC101927787 | 0.015830145 | 0.063514912 | 0.117767852 | 0.622889219 |
| FLG-AS1 | 0.026863428 | 0.074732754 | 0.104733866 | 0.689804887 |
